# Dermatologists’ perceptions of suicidality in dermatological practice: a survey of prevalence estimates and attitudes in Austria

**DOI:** 10.1186/s12895-020-00107-w

**Published:** 2020-09-29

**Authors:** Ekaterina Pronizius, Martin Voracek

**Affiliations:** 1 Department of Cognition, Emotion, and Methods in Psychology, Faculty of Psychology, University of Vienna, Vienna, Austria; 2 Wiener Werkstaette for Suicide Research, Vienna, Austria

**Keywords:** Suicidality, Suicide prevention, Mental health, Dermatology, Psychodermatology, Skin disorders

## Abstract

**Background:**

Chronic illnesses belong to suicide risk factors. The goal of the current study was to estimate the rate of suicide-related behaviors in patients with atopic dermatitis, psoriasis, or acne from a third-person perspective (namely, Austrian dermatologists).

**Methods:**

A link to a questionnaire specially developed for this study was emailed to 450 self-employed dermatologists in Austria, from which a total of 45 participated.

**Results:**

Three dermatologists reported more than five patients with atopic dermatitis, psoriasis, or acne who committed suicide in 2017. Seven doctors treated between 1 and 10 such patients suffering from suicidal ideation. These results are suggestive for a low rate of suicidal ideations in Austrian dermatology ordinations. The majority of dermatologists in the sample (82%) knew that these patients are at higher suicide risk. 60% of participants also believed that it rather would not be a problem for them to recognize suicidal ideation. When facing patients in a suicide crisis, reported intervention steps were: referring them to a specialist in psychiatry, or having a conversation about it. In the sample, most challenging about suicide was lack of time and lack of knowledge. Dermatologists were also interested in cooperating with mental health professionals and in the implementation of new prevention strategies (e.g., suicide-related training programs). Analysis revealed that private specialists, as compared with contract physicians, had fewer patients, but spent more time with them. Yet, these differences did not appear to influence the quality of treatment they provided. Treatment quality was defined as the extent to which doctors tell their patients that additional psychological treatments could be helpful and asking them about their emotional state. Female gender and a professional background in psychology impacted positively on treatment quality.

**Conclusions:**

Possible explanations for the low rate of suicidal ideations reported include the advanced Austrian health care system and dermatologists’ underestimation of the problem. Implications of the study are to promote cooperation between dermatologists and mental health professionals and to address patient suicidality from a first-person perspective (i.e., the patients).

## Background

Austria, similar to many other countries, is faced with a suicide problem. Currently, approximately 1200 of its citizens annually take their own life [1]. Various factors are associated with increased suicide risk, such as male gender or family history of suicide, as well as severe or chronic physical illness [2]. Skin disorders, such as atopic dermatitis, psoriasis, or acne, are chronic and incurable physical illnesses and therefore potentially are associated with increased suicide risk.

To reduce suicide prevalence, a national suicide prevention program (SUPRA) has been implemented. Alongside this, various research groups into suicidology exist, whose work is crucial for suicide prevention: the registered (and inter-university and interdisciplinary) research society Wiener Werkstaette for Suicide Research, the Austrian Society for Suicide Prevention, the Suicide Research Group at the Medical University of Vienna, and the Institute for Suicide Prevention and Research Graz. As for the field of psychodermatology, local and international professional associations exist: the European Society for Dermatology and Psychiatry, the Association of Psychoneurocutaneous Medicine of North America, and the *Arbeitskreis Psychosomatische Dermatologie der Deutschen Dermatologischen Gesellschaft* (working group Psychosomatic Dermatology of the German Dermatological Society).

The importance of suicide prevention programs is hard to underestimate. Suicide affects not only the victim and their close family members but also the whole community. Death by suicide or loss of productivity as a consequence of nonfatal suicide attempts is associated with great financial costs to the society [3].

Suicide experts [4] stress that the global numbers of deaths by suicide are much higher than those of the official statistic, since suicides are often underreported or misclassified as accidental. Official statistics also do not include nonfatal suicide attempts–suicide attempts that did not lead to death. The real number of suicide attempts is unknown. There are several reasons for this. First, a large number of suicide attempts stays unrecognized, unreported, and thus undocumented [3]. Second, suicide attempts not always lead to a contact with the healthcare system. International studies reveal that the real number of suicide attempts is 10–30 times higher than officially documented number of suicides [3].

### Skin disorders

Atopic dermatitis is a chronic or recurrent inflammatory skin disease [5]. It affects up to 20% of children and 1–3% of adults [5, 6]. In the majority of cases, atopic dermatitis has an early onset: 50% within the first year of life, and 30% between ages 2 and 7 [5, 7]. 85% of children with atopic dermatitis develop further atopic diseases until the age of 5 [5]. However, 70% of children with atopic dermatitis experience a spontaneous remission before adolescence [5]. Atopic dermatitis is a condition to which multiple factors are believed to be responsible for its onset and progression. These factors include genetic mutations, immune dysregulation, and environmental stressors, such as critical life events [8]. Characteristic symptoms of atopic dermatitis are skin inflammation, lichenification, dry skin, and persistent itch [4, 7]. Recent research has shown that atopic dermatitis is a systemic disorder and is associated with a higher risk for cardiovascular diseases, cancer, more frequent infections, and a higher risk for mental disorders [9]. The intensity of pruritus correlates positively with depression severity [4].

Psoriasis is “a chronic systemic disorder with characteristic sharply demarcated erythematous plaques” [4]. In the majority of cases (up to 75%), onset of psoriasis occurs between ages 30 and 40 [4]. Parisi et al. [10] carried out a systematic review of published population-based studies on the prevalence of psoriasis. This research revealed an increasing age trend and also showed cross-country differences in prevalence. Augustin et al. [6] found a prevalence of about 2% in a German population-based sample. As with atopic dermatitis, multiple factors, including genetics and environmental ones, are believed to be responsible for psoriasis onset and progression [10]. Considering the clinical severity of psoriasis (ranging from mild over moderate to severe), in mild cases individuals suffer from a few plaques, whereas in severe cases the disease is spread over the entire body [10]. Psoriasis may continually worsen with age or show exacerbations and remission episodes [10]. Psoriasis is additionally associated with multiple comorbidities, such as inflammatory bowel disease and major depressive disorder [4]. Around 30% of psoriasis patients suffer from psoriatic arthritis [4]. One multinational study showed that about 70% of psoriasis patients suffer from chronic itch [11].

Acne is a chronic inflammatory “disease of the pilosebaceous unit-hair follicles in the skin that are associated with an oil gland” [12]. Acne prevalence differs between countries: from 3.9% in Germany to 8.1% in China [13]. The onset of this disease mostly occurs during adolescence and to some degree affects a vast majority of teenagers [around 85% between ages 12 to 24 years; 4]. Other studies observed a much lower prevalence of acne in adolescence, namely, about 25% in the age group of 16–20 years [6]. These differences in acne prevalence may result from the fact that mild cases of acne can easily be overlooked [13]. Other reasons are possible definitional and diagnostic differences and true variability in the prevalence of these disorders [13]. Even though acne often is referred to as a skin condition, mostly affecting young adolescents, it can well persist into adult age [6]. However, acne generally shows a decreasing age trend: from 10% in the age group 21–30 years, over 5% in the age group 31–40 years, to merely 2% beyond the age of 40 years [6]. As with atopic dermatitis and psoriasis, multiple factors are believed to be responsible for acne onset and progression. These include a diet with a high glycaemic index, genetic factors, and the *Propionibacterium acnes* bacterium [13]. Other factors that have been suggested include the menstrual cycle, picking, and emotional stress [12]. Symptoms of acne are seborrhea, both non-inflammatory and inflammatory lesions, and various degrees of scarring [12]. Acne usually affects the face, neck, upper chest, shoulders, and back [12]. Several severity grading scales for acne have been devised. One of these is a three-grade scale (with grades mild, moderate, and severe) “based on the presence of comedones, papules/pustules, and nodules, respectively” [13].

Dermatology is often described as a field with “primarily an outpatient-oriented specialty, most of the skin diseases being associated with variable morbidity but low mortality” [14]. Chronic skin conditions have a massive impact on quality of life, such as restricting individuals to some degree in fulfilling their social roles [7, 11, 15–20]. Blemished skin causes embarrassment, shame, and often is a reason for low self-esteem [17]. Individuals with chronic skin conditions are often bullied at school, have problems with social functioning, and are stigmatized [17]. As a consequence, chronic skin conditions may cause depression, which in turn, increases the risk of suicidal ideation, suicide attempts, or suicide itself [2, 17]. About 30% of dermatology patients suffer from some form of mental disorder [4].

Picardi et al. [21] reported prevalence estimates for suicidal ideations in psoriasis (10%) and acne (7%), with a total prevalence of almost 9% among patients with common dermatological conditions. In contrast, a retrospective case-control study from the USA [22] found no significant association between skin conditions (e.g., atopic dermatitis, psoriasis, and acne) and suicide-related behaviors.

New treatment approaches recently emerged. One of them – a psychodermatological approach – uses psychological methods in the treatment of some dermatological conditions. The postulated close link between skin and mind is based on the fact that both the skin and nervous system share a common developmental origin, namely, the embryonic ectoderm [23]. Nonpharmacologic methods reported as promising in treating chronic skin conditions include biofeedback, cognitive-behavioral methods, and hypnosis [24, 25].

According to [26], most psychodermatological conditions can be grouped into four categories: psychophysiological disorders, primary and secondary psychiatric disorders, and cutaneous sensory disorders. Psychophysiological disorders are conditions wherein skin inflammation is exacerbated by emotional stress. Primary psychiatric disorders are characterized by absence of primary skin diseases (e.g., trichotillomania, delusions of parasitosis). Skin manifestations, if any, are self-induced. Patients with secondary psychiatric disorders develop emotional problems as a result of their skin condition. Finally, cutaneous sensory disorders are disorders with a purely sensory complaint, without visible evidence of skin conditions [26].

Atopic dermatitis, psoriasis, and acne can all be characterized as both psychophysiological and secondary psychiatric disorders [26], thus leading to a vicious circle: stress or critical life-events can cause worsening of the skin condition; vice versa, this can lead to psychiatric disorders, such as depression [20, 27]. To break this vicious circle of stress-induced worsening of the skin, psychological or psychiatric aspects in the treatment of these particular skin conditions are needed.

In their review, [2] addressed the issue of suicide risk in skin disorders (e.g., psoriasis, acne, and atopic dermatitis). These researchers postulated that dermatologists could help in recognizing and preventing suicides in these patient groups. The suicide prevention plan of [2] includes questionnaire-based depression screening (because depression is associated with increased suicide risk) and suicide-centered dialogue. Screening questionnaires for depression need to be brief and validated for dermatological settings. Picardi et al. [2] provide examples of such questionnaires: the Patient Health Questionnaire [28, 29] and the Primary Care Screener for Affective Disorder [30].

In the course of a suicide-centered dialogue, dermatologists can directly ask their patients about suicidal ideation or previous suicide attempts [2]. During the whole conversation, the dermatologist should remain calm, empathic, and nonjudgmental [2]. The suicide-centered dialogue should start with milder questions, addressing the emotional well-being of the patient. If the patient expresses negative feelings, the dermatologist might continue with asking about death wishes in general. In case the patient confirms their desire to escape life, detailed questions about specific suicide plans are required. The main goal of the dermatologist is to estimate the imminence of suicidal behavior. Based on this estimation, the immediate actions of the dermatologist could be: (a) referring the patient to a mental health professional, (b) alerting patient’s close relatives, (c) carrying out an emergency psychiatric evaluation in case of a high suicide risk, (d) providing a supervision of the patient, (e) hospitalization, (f) removal of potential methods of suicide, and (g) initiation of treatment of associated psychiatric disorders [2].

However, it is unknown whether dermatologists in Austria share the similar opinions regarding suicide prevention in their patients. If so, it would be crucial to find out how such psychological elements can be implemented in the everyday life of the dermatological practices. We therefore set out to estimate the feasibility of implementing the described prevention plan of [2] from a dermatologists’ point of view. Picardi et al. [21] state that such prevention steps would not lengthen doctor visits and would increase the efficiency of the consultation. Moreover, the prevention of suicide in these groups of patients strongly depends on the successful cooperation between dermatologists and mental health professionals [2]. We aimed to clarify whether additional support on the part of mental health professionals is really desired.

### Study aims

The main aim of this study was to estimate the rates of suicides, suicide attempts, and suicidal thoughts in Austrian patients with atopic dermatitis, psoriasis, or acne, based on the third-person perspective of dermatologists’ self-reports.

The second study aim was to assess knowledge with regards to suicide and suicide intervention or prevention strategies among Austrian dermatologists. Finally, through this endeavor we aimed to contribute to promoting cooperation between dermatologists and mental health professionals. An additional goal was to test whether gender, length of work experience, contract with insurance companies (vs. none), and psychological knowledge of dermatologists influence the treatments and interventions they provide.

With our online survey study, we parallel aimed to fulfill three strategic SUPRA goals: strategic objective 2 (support and treatment), 4 (awareness and knowledge), and 6 (quality assurance and expertise). The SUPRA program stands on six equal pillars of suicide prevention. The other three pillars are: (a) coordination and organization, (b) restriction of suicide means, and (c) embedment in prevention and health promotion programs [3].

### Health-care system in Austria

In Austria, outpatient care is mostly provided by self-employed or resident doctors (in German: *niedergelassene Ärztinnen und Ärzte*) running their own medical practices [31]. Based on the payment of medical bills, the group of the self-employed doctors can further be subdivided into three distinct subgroups (see Table 1): contract physicians and two types of private specialists (*WahlärztInnen* and *PrivatärztInnen*).

**Table 1:** Health Care System in Austria: Key Terms

	Self-employed/resident doctor			Employed doctor
	Contract physician	Private specialist	Contract/private dermatologist	
German term	*KassenärztInnen*	*Privat- und WahlärztInnen*	*Alle Kassen und privat*	*Angestellte ÄrztInnen*
Working conditions	contract with SHI Fund	working privately	contract with SHI Fund and working privately	contract with SHI Fund and/or working privately
Distinctive features	treatments directly reimbursed by SHI Fund; longer waiting time for appointment and shorter duration of doctor’s consultations	not or only partially reimbursed by SHI Fund; shorter waiting time for appointment and longer duration of doctor’s consultations	combines features of contract and private practices	provides inpatient care (employment in a hospital)

*Note*. *SHI Fund* Social Health Insurance Fund

There, of course, exists another group of doctors, the so-called employed doctors (*angestellte Ärztinnen und Ärzte*). These provide inpatient care in hospitals. Some of these doctors are exclusively working in a hospital; others are running a practice of their own as well. This former group (employed dermatologists without own practice) was not eligible for study participation.

Contract physicians (*KassenärztInnen*) are medical doctors, who signed a contract with the Social Health Insurance Fund (SHI Fund, [32]). Medical services, which are directly reimbursed by the SHI Fund are explicitly specified, but vary between different health insurance companies. Even though most of treatments and medications are free of charge for insured patients, the ordinations of contract physicians are reported to be overcrowded, with long waiting times for an appointment [33]. Consequently, a doctor’s examination usually lasts only a few minutes [33].

Private consultants (*WahlärztInnen*) are medical doctors who did not sign a contract with any health insurance company [32]. Patients have to pay the entire medical fees directly [32]. A part of the costs can be reimbursed afterwards by a health insurance company [32]. Patients of private physicians (*PrivatärztInnen*) also have to pay all medical fees directly, and no costs are reimbursed by health insurance companies [34]. To avoid any confusion, in this study private consultants and private physicians were merged into a single group of private specialists, which is characterized by shorter waiting times for appointments and longer duration of doctor’s consultations, as compared to contract physicians [33].

Some doctors are working privately and have a contract with an insurance company as well (*privat* and *alle Kassen*). We termed this group as contract/private dermatologists.

Conceivably, the aforementioned differences between contract physicians and private specialists in terms of consultation time they typically spend with their patients may well (but do not necessarily) influence treatment and intervention outcomes.

### Research hypotheses

In the first place, this study was descriptive and explorative. Nonetheless, we also formulated several testable hypotheses that aimed to confirm or to reject empirical findings of recent prior related research stemming from other countries than Austria.

#### Hypothesis 1: contract with an insurance company as a factor

On average, private specialists are believed to spend more time with their patients and thus have fewer patients than contract physicians. We explored whether this was observable in our sample. Moreover, such differences in terms of time spent and the number of patients could impact treatments.

We therefore formulated the following set of directional hypotheses:

- H1a: Private specialists have fewer clients than contract physicians.
- H1b: Private specialists on average spend more time with their patients than contract physicians do.
- H1c: Private specialists tell their patients more often than contract physicians that additional psychological, psychotherapeutic, or psychiatric treatments could be helpful.
- H1d: Private specialists more often ask their patients about their emotional state than contract physicians do.

#### Hypothesis 2: gender as a factor

Gender of physicians appears to be an important variable that should be accounted for. Meta-analytic evidence suggests several gender effects in medical communication [35]; for example, that female primary care physicians are working more patient-centered than their male counterparts. This includes being engaged in more communications and having longer visits [35]. Based on this evidence, we decided to formulate two directional hypotheses:

- H2a: Female doctors more often than male doctors tell their patients that additional psychological, psychotherapeutic, or psychiatric treatments could be helpful.
- H2b: Female doctors more often than male doctors ask their patients about their emotional state.

#### Hypothesis 3: work experience as a factor

Length of work experience could have an impact on treatment [36, 37]. We are unaware of matching studies that would allow to formulate directional hypotheses. Hence, the following hypotheses were formulated:

- H3a: Length of work experience has an influence on how often doctors tell their patients that additional psychological, psychotherapeutic, or psychiatric treatments could be helpful.
- H3b: Length of work experience has an influence on how often doctors ask their patients about their emotional state.

#### Hypothesis 4: background in psychology as a factor

Some skin conditions, such as atopic dermatitis, psoriasis, and acne, should be treated using psychotherapeutic elements. We expected that doctors with a background in psychology would not only be aware of this fact, but would also seek to improve their patients’ mental health skills. Consequently, they might advise their patients to start an additional psychological treatment more often than doctors without a background in psychology. We therefore hypothesized:

- H4a: Doctors with a background in psychology more often tell their patients than doctors without such a background that additional psychological, psychotherapeutic, or psychiatric treatments could be helpful.
- H4b: Doctors with a background in psychology more often ask their patients about their emotional state than doctors without such a background.

## Methods

### Online vs paper-pencil

Conducting a survey, we had to choose between online or paper-pencil versions. We decided to develop an online survey, based on several reasons: first, economic advantages; second, efficiency of the data collection [38]; and third, reduced environmental costs.

There are some problems typical for online surveys that are also a matter of concern for our study. On the other hand, our population of interest is relatively small (there are not that many dermatologists in Austria), so we could try to avoid or minimize some errors; for example, a coverage error “in which all units of a population do not have an equal probability of inclusion in the sample” [38]. Almost 10 years ago, a major challenge concerning online survey was that not all parts of the population had an access to the internet [Schaefer & Dillman, 1998 as cited in 38]. Nowadays, the internet is an inseparable part of our life. Before choosing between an online and paper-pencil version of our questionnaire, our online search revealed that the vast majority of dermatologists in Austria published their own e-mail address on the internet.

Another error, sampling error occurs, when “only a portion of the sample rather than all members” are being studied [38]. A good way to reduce the sampling error is to increase the number of participants [38]. We were trying to use different techniques that could help to increase the response rate. Examples of these techniques are offering incentives, developing a respondent-friendly layout, sending reminders, contacting dermatologists via telephone, etc. However, a small response rate, especially for web surveys, is known to be a major obstacle [38].

Of course, a paper-pencil version of a questionnaire is in some aspects more attractive for the participants. They can skip questions or answer the questions how they want. On the other hand, during the data analysis, it can be very challenging to work with an incomplete data. An implementation of a questionnaire online allows the researcher to prohibit such participants´ behavior. Based on these considerations, we developed an online questionnaire and intentionally decided not to permit the skipping of questions. However, such restrictions might decrease the participation motivation and increase the drop-out rate.

There has been, in some aspects, a similar study done in Vienna, Austria in 2002. Ritter et al. [39]. developed an explorative questionnaire, with which they investigated the suicide risk-related knowledge and attitudes of general practitioners of Vienna. This explorative questionnaire was sent to a sample of 200 randomly chosen doctors by post. An additional call was made, to ensure that all general practitioners received an envelope. The response rate was 33% (*n* = 66). With our study, we would be able to compare the response rate between our online survey and the postal survey of [39].

The duration of the questionnaire is another very important issue. The rule of thumb dictates that “the longer the questionnaire is, the less likely people will participate” [Sheehan & McMillan, as cited in 38]. Initially, the length of the pretest version of our questionnaire was quite long (up to 20 min).

Van Selm et al. [38] describe three main ways of distributing questionnaires online: (a) sending respondents an e-mail with an attached questionnaire, (b) emailing respondents an introductory letter with a hyperlink to the questionnaire, and (c) placing a link on a web page. Sending respondents the entire questionnaire via e-mail (a) would not allow us to conduct an anonymous survey since we would see from what e-mail address the document is returned. Exploring such a sensitive topic as suicidality would profit when anonymity is guaranteed to the participants [40]. Placing a link on a web page (c) would involve a risk that someone else and not the participants from the population of interest would participate in our study. Therefore, we decided to email respondents an introductory letter with a hyperlink to the questionnaire (b). To increase the response rate, we sent only personalized e-mails.

As for incentives, we offered dermatologists to let their e-mail address, if they want to receive the results of the study. To maintain anonymity and confidentiality, these e-mail addresses were stored separately from the collected data. Initially, we decided not to add any other incentives, such as payment for participation. However, we were ready to offer a one euro donation per participation to any non-governmental organization, in case it comes to a low response rate. On the last page of our questionnaire, we placed our contact e-mail address, so that participants could contact us anytime they are experiencing troubles, or have unresolved issues regarding the topic of the study.

We implemented the questionnaire on [41]. This particular platform has many advantages, such as export of the data in Microsoft Excel- and SPSS-compatible format, data visualization, filter questions, online support, the option to save data page by page, and many more.

### Online survey form

The online questionnaire specifically developed for this study comprised 18 single-choice and 10 multiple-choice questions, plus one open-ended question (totalling 29 questions, Additional file 2: Appendix A). Estimated survey completion time amounted to up to 10 min.

Some of the single-choice (and all of the multiple-choice) questions were accompanied by an additional open-ended answer category. These questions are 3, 4.1, 8, 10, 11, 12.1, 13.1, 17, 18.1, 19.1, 21, 22, and 23. For the two content analyses (Q. 22 and Q. 23), we reviewed and categorized the individual responses provided by participants three times, with a seven-day interval between. The initial coding scheme was data-driven. Appropriateness of the final categories was additionally checked by a second independent rater. Since the categories are not mutually exclusive (one open-ended response of a doctor could simultaneously fall into more than one category), we refrained from attempting to quantify interrater agreement.

#### Part I

Basic sociodemographic information was queried in the first survey part. Data confidentiality and anonymity is important for investigating sensitive topics such as suicidal behavior. Consequently, only a minimum of questions regarding sociodemographic characteristics were placed into the survey. Socio-demographic data included gender (Q. 1), length of the work experience (Q. 2), and with which insurance company physicians signed a contract (Q. 3). Based on these answers we created a new variable *contract with an insurance company* with three variable categories: contract physicians, contract/private dermatologists, and private specialists. Dermatologists were referred to the group of contract physicians if they chose any of the insurance companies (GKK, BVA, SVA, VA, KFA, and/or SVB) and no “private” answer category. Private specialists include dermatologists, who chose the “private” answer category and did not choose any of the insurance companies. And dermatologists, who chose both, any of the insurance companies as well as the “private” answer category, were referred to the group contract/private dermatologists.

Further questions targeted whether physicians have a psychology-related theoretical background: certain psychological diplomas (Q. 4.1), degrees in psychology (Q. 5 and Q. 6), membership in psychodermatology working units (Q. 8), or a participation in suicide training programs within the last 5 years (Q. 7). We created a new dichotomous variable psychological background with two variable categories - psychological background “yes” or “no”. To the psychological background “yes-group”, a dermatologist was referred, if they answer positively on at least one of the questions described above (Qs. 4.1, 5, 6, 7, and 8).

The 9th question asks if skin doctors are familiar with the Austrian suicide prevention program SUPRA.

In the two last questions, doctors had to provide an estimation of the average amount of patient visits a week (Q. 10) and the average duration of such visits (Q. 11). To perform any statistical tests, we had to prepare the data from the group of contract/private dermatologists. They provided two answers to one question: “How many patient contacts do you have as contract physicians and as private specialists?”. We added these values together. By doing so, we got a total number of patient contacts that contract/private dermatologists are treating a week. Consequently, some information (e.g. distinction between private and contract visits) got lost. This loss of data had to be taken into account, since it allows conducting a comparison of group differences using conventional tests, for example, a Kruskal-Wallis H test. Similarly, the group of contract/private dermatologists provided two estimates of the average duration of their doctor consultations. Here, we built a mean of these two values. By doing so, we got a mean duration of doctor consultations, but at the same time lost the distinction between private and contract consultations.

During the data analysis, we were faced with an inconsistency in the answers of eight dermatologists. In the third question, they needed to choose whether they work privately, have a contract with an insurance company or both. Later they were asked to provide an estimate of the average amount of patient visits a week (Q. 10) and the average duration of such visits (Q. 11). Some dermatologists who, for example, first said that they work solely as contract dermatologists, then provided an estimation of the average amount of contract and private patient visits a week. Surprisingly, those dermatologists provided a non-negligible number of “forgotten” patients. This unexpected inconsistency in answers had an impact on the data analysis.

Based on the answers to the questions 10 and 11, we created a new variable *contract with an insurance company2*, with the same variable categories contract physicians, contract/private dermatologists, and private specialists. This new variable was included in all subsequent relevant steps of the data analysis. The reason for this is that the questions 10 and 11 provide more information than question 3 and have therefore more weight for the data analysis.

#### Part II

In the second survey part, we queried estimates regarding dermatologists’ recalled number of completed suicides (Q. 14), suicide attempts (Q. 15), and suicidal thoughts (Q. 16) in their patients with atopic dermatitis, psoriasis, and acne. These skin disorders are standing in focus of our research. They were not chosen arbitrarily. First, without specifying the particular skin illnesses, it would be challenging for dermatologists to answer suicide-related questions. There is a wide range of dermatological skin conditions, that all are characterized by their own uniqueness and mortality rate. On the other hand, for the purpose of broader generalization of our results, we did not want to narrow our questionnaire to only one specific disorder. As it was shown in the introduction, atopic dermatitis, psoriasis, and acne are sharing some major features and are all associated with an increased suicide risk.

At the beginning of the second survey part we asked, whether dermatologists know that patients with atopic dermatitis, psoriasis, and acne are at a higher risk of suicide (Q. 12) and more often than a control group are suffering from suicidal thoughts (Q. 13). Answering “yes” on these two questions, dermatologists had to specify where they learned this information (Q. 12.1 and 13.1, respectively). The second part ended with the question about intervention strategies that dermatologists would or are attempting when treating patients at a higher suicide risk (Q. 17).

#### Part III

The focus of the third part was on the interactions of mind and skin. We operationalized the quality of treatment in our study as whether dermatologists (a) tell their patients that an additional psychological, psychotherapeutic or psychiatric treatment could be helpful (Q. 18); and (b) ask their patients about their emotional state (Q.19). If they don’t discuss these two issues with their patients, they had to specify the reasons why (Q. 18.1 and Q. 19.1, respectively). In the 20th question dermatologists needed to rate how difficult it would be for them to recognize suicide intentions in their patients. With the last question of the third part (Q. 21) we wanted to find out, what is the dermatologists’ biggest challenge regarding suicide in patients with chronic skin conditions.

#### Part IV

The fourth and last part comprised items about suicide prevention. Austrian dermatologists were asked to appraise the intervention of [2; Q. 22], described as follows:

Picardi et al. [2, 21] suggest that dermatologists could help to prevent suicide in patients with chronic skin conditions by providing them with brief depression questionnaires (such as Patient Health Questionnaire PHQ). If such depression screening is not possible, the physician could directly inquire about it.

We also wanted to know, if and how clinical psychologists or psychotherapists can assist dermatologists in their work (Q. 23). The last question of the fourth part asked whether dermatologists are wishing for more suicide-related training programs (Q. 24).

All survey questions were derived from the literature review and the key findings of recent topically relevant empirical studies, as elaborated on and discussed above (Background).

### Research hypotheses: Statistical tests

#### Hypothesis 1: contract with an insurance company as a factor

In our sample, we had three groups of participants: contract physicians, contract/private dermatologists, and private specialists. To test whether there are statistically significant differences between these three groups, we could perform a one-way ANOVA or, alternatively, Kruskal-Wallis *H* tests, along with Jonckheere-Terpstra tests (done here, because ANOVA assumptions were not met).

- H1a: Private specialists have fewer clients than contract physicians.

The a priori assumption about the order of the groups for the Jonckheere-Terpstra trend test:

$$ {Mean\ ranks}_{contract}\ge {Mean\ ranks}_{\mathrm{contract}/\mathrm{private}}\ge {Mean\ ranks}_{private} $$

During our online search, we found information that private specialists are assumed to have fewer patients than contract physicians. However, the decision to place the group of the contract/private dermatologists into the middle of the Equation was based on the assumption that is not proven empirically. The assumption is that the group of contract/private dermatologists combines in itself features of the two other groups. Whereas private specialists are believed to have fewer patient contacts than contract physicians do, the group of contract/private dermatologists is therefore expected to lay somewhere between.

- H1b: Private specialists on average spend more time with their patients than contract physicians do.
- H1c: Private specialists tell their patients more often than contract physicians that additional psychological, psychotherapeutic, or psychiatric treatments could be helpful (on a scale ranging from 0 *= never* to 3 *= almost on all occasions*).
- H1d: Private specialists more often ask their patients about their emotional state than contract physicians do (on a scale ranging from 0 *= never* to 3 *= almost on all occasions*).

H1b – H1d. The a priori assumption about the order of the groups for the Jonckheere-Terpstra trend test:

$$ {Mean\ ranks}_{contract}\le {Mean\ ranks}_{\mathrm{contract}/\mathrm{private}}\le {Mean\ ranks}_{private} $$

H1a:H1d: If the Jonckheere-Terpstra trend test reveals non-significant results and given the fact that the study is explorative, we additionally calculated the Kruskal-Wallis H test, in order to test whether there are any between-group differences.

#### Hypothesis 2: gender as a factor

- H2a: Female doctors more often than male doctors tell their patients that additional psychological, psychotherapeutic, or psychiatric treatments could be helpful (on a scale ranging from 0 *= never* to 3 *= almost on all occasions*).
- H2b: Female doctors more often than male doctors ask their patients about their emotional state (on a scale ranging from 0 *= never* to 3 *= almost on all occasions*).

H2a – H2b. The a priori assumption for the Mann-Whitney U test:

$$ Mean\ \mathit{\operatorname{rank}}{s}_{females}>{Mean\ ranks}_{males} $$

For testing this hypothesis, we chose the Mann-Whitney U test. The Mann-Whitney U test is a nonparametric alternative to the t-test, which allows comparing differences between two independent groups.

#### Hypothesis 3: work experience as a factor

- H3a: Length of work experience has an influence on how often doctors tell their patients that additional psychological, psychotherapeutic, or psychiatric treatments could be helpful (on a scale ranging from 0 *= never* to 3 *= almost on all occasions*).
- H3b: Length of work experience has an influence on how often doctors ask their patients about their emotional state (on a scale ranging from 0 *= never* to 3 *= almost on all occasions*).

H3a – H3b. The a priori assumption for the Kruskal-Wallis H test:

$$ Mean\ {\mathit{\operatorname{rank}}}_1\ne Mean\ {\mathit{\operatorname{rank}}}_2\ne Mean\ {\mathit{\operatorname{rank}}}_3\ne Mean\ {\mathit{\operatorname{rank}}}_4 $$

The a priori assumption for the Jonckheere-Terpstra trend test (explorative):

The natural order of the groups (10 years or less, 11–20, 21–30, and 31 years or more), without specifying a decreasing or increasing trend across these ordered groups.

#### Hypothesis 4: background in psychology as a factor

- H4a: Doctors with a background in psychology more often tell their patients than doctors without such a background that additional psychological, psychotherapeutic, or psychiatric treatments could be helpful.
- H4b: Doctors with a background in psychology more often ask their patients about their emotional state than doctors without such a background.

H4a – H4b. The a priori assumption for the Mann-Whitney *U* test:

$$ {Mean\ ranks}_{with}>{Mean\ ranks}_{with out} $$

### Sampling frame

For Austria, several online databases exist which provide contact information of currently practicing physicians (*ÄrztInnenverzeichnis* or medical register). For the recruitment of participants, we utilized the internet database [42], along with the internet databases provided by the medical associations of each of the 9 federal states of Austria (Additional file 2: Appendix B1).

Keywords for searching in these databases were *Facharzt für Haut- und Geschlechtskrankheiten* (doctor for skin and venereal diseases), *Dermatologie* (dermatology), and *niedergelassene ÄrztInnen* (doctors running their own practice, i.e., the so-called self-employed or resident doctors). Exclusion criteria were not being listed on the homepage of the Austrian medical association and not having an own practice (employed dermatologists or *angestellte ÄrztInnen*). Doctors both working at a hospital (employed dermatologists) and running their own practice (self-employed doctors) were invited to participate in the survey. These searches yielded a total of 716 results (Additional file 2: appendix B2; note that some dermatologists may run their practice simultaneously in more than one federal state within Austria; therefore, the number of unique results is smaller than 716)

Our sampling methodology was based, therefore, on the voluntary participation of a large majority of the total population of dermatologists in Austria (specifically, only those dermatologists were invited whose email was found one the internet)

### Data collection

Data collection lasted from Feb 12 to Apr 8, 2018 (Additional file 2: Appendix B3).

#### Vienna

Two hundred twenty-five dermatologists of Vienna–more precisely their administrative staff–received a call from us. We introduced ourselves and asked permission to send an e-mail with a link to the questionnaire. If permission was granted, the e-mail was sent. From these 225 dermatologists, 58 refused directly on the telephone or per e-mail. Twelve refused to confirm their e-mail address, although, offered us to send them the questionnaire by post. That was implemented on the 15th of March 2018. We mailed 12 questionnaires together with stamped addressed envelopes. Two filled-out questionnaires came back.

#### Other federal states of Austria

During the sample recruitment in other federal states, we decided to send e-mails without contacting the practice administrative staff first. Sending e-mails without previous calling was done intentionally because of several reasons. First, taking Vienna as an example we saw, that many refuses came within the first seconds of the telephone conversation and thus we did not have a possibility to tell more about the project. Second, the response rate in Vienna was extremely low, and a telephone call did not seem to increase the participation motivation. Even an adverse effect of the telephone call is possible, where the previous calling is decreasing the participation motivation. Third, when sending questionnaires by post, it didn’t lead to a much higher participation rate but increased the environmental and financial costs.

Together with Vienna, we sent 438 e-mails and 12 questionnaires by post (236 female doctors and 214 male doctors). At the end of these data collection waves, we had 24 fully completed questionnaires.

#### Sending the reminders

Before sending the reminders, we decided to change the text of the invitation hoping that this would increase the response rate. We also added, that for each participation we would donate one euro to the non-governmental organization Doctors Without Borders.

On the 20th of March, we started sending the reminders. The last reminders were sent on the 27th of March. In total, we sent 455 reminder e-mails (Additional file 2: Appendix B3). In addition, one colleague disseminated the link to the participants of the 5th Workshop of the working group *Psychodermatologie* (Mar 9–10, 2018; Goldegg, Salzburg, Austria). These efforts combined yielded 43 completed questionnaires which were returned.

#### The response and completion rates

When calculating the response rate, we considered the partial interviews as responders if the questionnaire was filled out at least till page 7. The number of all completed surveys (till final page 9) and the number of the partial surveys that met the aforementioned conditions were placed in the numerator. In the denominator stands the mean number of valid invitations, we sent during all data collection waves.

$$ Response\ rate=\frac{\mathrm{Number}\ \mathrm{of}\ \mathrm{completed}\ \mathrm{surves}+\mathrm{number}\ \mathrm{of}\ \mathrm{partial}\ \mathrm{surveys}}{\mathrm{Mean}\ \mathrm{number}\ \mathrm{of}\ \mathrm{invitations}\ \mathrm{sent}}\times 100\% $$

The completion rate was calculated by placing the number of the completed (9 pages) surveys in the numerator. In the denominator stands the number of all responders who entered the survey: fully completed surveys, partial interviews (min. 7 completed pages), and interviews with fewer than 7 completed pages.

$$ Completion\ rate=\frac{\mathrm{Number}\ \mathrm{of}\ \mathrm{completed}\ \mathrm{survey}\mathrm{s}}{\mathrm{Number}\ \mathrm{of}\ \mathrm{respondents}\ \mathrm{who}\ \mathrm{entered}\ \mathrm{the}\ \mathrm{survey}}\times 100\% $$

## Results

Forty-three dermatologists filled out the questionnaire till the final page. One participant exclusively was an employed doctor and thus excluded from the sample. Three additionally returned questionnaires were to some extent incomplete, but included in analysis. Incomplete surveys with less than 7 pages were excluded. The final sample size for analysis therefore amounted to 45 respondents.

The response rate was 10%.

$$ Response\ rate=\frac{43+3}{\left(450+455\right)/2}\times 100\%=10.17\% $$

The total completion rate of the survey was approximately 75%.

$$ Completion\ rate=\frac{43}{\left(43+3+11\right)}\times 100\%=75.43\% $$

As a result, the final sample size is (a) *N* = 45 (18 females) for the questions 1–12 and 14–21, (b) *N* = 44 for the question 13, (c) *N* = 41 for the question 22, and (d) *N* = 42 for the questions 23–24.

### Descriptive sample statistics

#### Part I. Sociodemographic data

More male than female doctors participated in the study (27 men, 18 women). However, more women than men received an invitation for study participation (236 women, 214 men). Therefore, the gender composition of the sample differed somewhat from that of the population of interest. However, a nonresponse bias analysis with regards to gender was not significant (binomial test: *p* = .07, one-tailed).

Length of work experience was similarly distributed across groups. Fourteen doctors reported running their practice for 10 years or less, 13 doctors for 11–20 years, followed by groups with 21–30 years of experience (*n* = 12), and 31 or more years of experience (*n* = 6).

The majority of dermatologists (*n* = 26) had no contract with an insurance company and were working privately; eleven dermatologists had signed a contract with at least one insurance company; and eight doctors were contract/private dermatologists.

In response to the questions about an additional professional background in psychology, four doctors answered that they had an ÖÄK (*Österreichische Ärztekammer*) diploma in psychosocial medicine (PSY-I) and five doctors that they had the (higher-level) ÖÄK diploma in psychosomatic medicine (PSY-II). None of the doctors had the (highest level) ÖÄK diploma in psychotherapeutic medicine (PSY-III). Seven dermatologists reported being members of a psychodermatology working unit. Another two physicians visited suicide-awareness or intervention training programs over the past 5 years. One doctor had visited seminars on the psychology of chronic patients, and one doctor concurrently was a psychotherapist (Q. 4.1, open-end). All other questions related to a psychological background were negatively answered by all participants, including the question: “Do you additionally have a psychology degree/degree in clinical psychology?” For further analysis of psychological background, we created a new dichotomous variable (yes vs. no; 11 vs. 34 respondents). Descriptive data of the dermatologists with a psychological background are in Additional file 2: Appendix C1).

The majority of the sample (*n* = 36) had never heard about the Austrian suicide prevention program SUPRA, whilst nine doctors said that they had heard something about it.

Sociodemographic data of the sample are summarized in Table 2.

**Table 2:** Sociodemographic Sample Data

Variable	*n*
Gender	
Male	27
Female	18
Length of work experience	
10 years or less	14
11–20 years	13
21–30 years	12
31 years or more	6
Contract with an insurance company2^a^	
Contract physicians	11
Contract/private dermatologists	8
Private specialists	26
Psychological background	
Yes	11
No	34
SUPRA	
Slightly familiar with the program	9
Never heard about it	36

*Note*. *N* = 45. *n* = number of participants

^a^Based on the answers to the questions 10 and 11

As expected, the average number of patient contacts and the duration of doctor’s consultations differed between contract physicians, contract/private dermatologists, and private specialists (Table 3).

**Table 3:** Average Number of Patient Contacts and Average Duration of Doctor’s Consultations

Variable	*n*	*M (SD)*	95% *CI* for *M*
Number of patient contacts			
Contract physicians	11	280.00 (139.14)	[186.52, 373.48]
Contract/private dermatologists^a^	8	161.88 (89.28)	[87.23, 236.52]
Private specialists	26	61.27 (62.38)	[36.07, 86.47]
Duration of doctor’s consultations^b^			
Contract physicians	11	8.32 (2.98)	[6.31, 10.32]
Contract/private dermatologists^c^	8	16.25 (5.00)	[12.07, 20.43]
Private specialists	26	24.88 (7.40)	[21.90, 27.87]

*Note*. *N* = 45. *n* number of participants; *M* mean; *SD* standard deviation; *CI* confidence interval

^a^Sum of contract and private patient contacts. ^b^In minutes. ^c^ Average duration of contract and private patient consultations

#### Part II. Suicide risk in patients with chronic skin conditions

The majority of doctors knew that patients with atopic dermatitis, psoriasis, or acne are at a higher suicide risk (*n* = 37). This information primarily came from training programs (*n* = 30), followed by deliberate research (*n* = 11), experience (*n* = 5), accidental discovery (*n* = 4), or from their colleagues (*n* = 3).

In similar vein, the majority of doctors knew that patients with atopic dermatitis, psoriasis, or acne more often suffer from suicidal thoughts than control groups (*n* = 34). Similar to the above question about suicide, this information primarily came from training programs (*n* = 28), followed by deliberate research (*n* = 9), experience (*n* = 7), from their colleagues (*n* = 4), or from accidental discovery (*n* = 3).

Questions related to the number of suicides in doctors’ practices were almost throughout answered in the negative. Thirty-seven doctors said that they had no patients with atopic dermatitis, psoriasis, or acne who committed suicide within the previous 12 months. Five doctors said that they did not know how many of their patients committed suicide in the past year. Although, three participants said, that they had more than five such patients.

Similar answers were given to the question about suicide attempts: thirty-three doctors said that they had no patients with atopic dermatitis, psoriasis, or acne who attempted suicide in the past year. Twelve skin doctors stated they did not know. Thirty-six doctors stated that during the previous 12 months, none of their patients with these skin conditions expressed suicidal thoughts, and two doctors could not provide an estimate. Seven doctors said that within the last year they treated 1–10 patients with atopic dermatitis, psoriasis, or acne who suffered from suicidal thoughts.

Reported intervention steps of dermatologists in case of facing patients at acute suicide risk included: referral to a specialist in psychiatry (*n* = 38), having a conversation about it (*n* = 33), involving relatives (*n* = 16), referral to a psychiatric ambulance or clinic (*n* = 15), arranging a new appointment with the patient (*n* = 15), recommending crisis intervention centers (*n* = 14), and prescribing psychiatric medication (*n* = 1).

#### Part III. The interaction between mind and skin

Twenty-six doctors said that they rarely tell their patients that psychological, psychotherapeutic, or psychiatric treatments could be helpful in treating their skin conditions; sixteen doctors said this frequently; and two skin doctors reported to talk about this with their patients on all occasions. One participant wrote that s/he never tells patients that psychological, psychotherapeutic, or psychiatric treatments could be helpful in treating skin conditions, stating that suicide-related topics are not part of the dermatologist’s job.

Twenty-four doctors asked their patients frequently about their emotional state, eleven physicians did it on almost all occasions, and ten doctors rarely asked their patients about their emotional state. None of the doctors selected the option “I never ask the patients about their emotional state”.

In response to the question of whether dermatologists would have troubles in recognizing suicidal intentions in their patients, twenty-five skin doctors chose the answer “rather no”, sixteen doctors said “rather yes”, two dermatologists answered “no”, and two dermatologists stated “yes, they would have troubles in recognizing suicide in their patients”.

For the doctors, most challenging about suicide was the lack of time (*n* = 18), followed by lack of knowledge (*n* = 15), the notion that suicide-related subjects are not part of their job (*n* = 10), problems to establish contact with patients (*n* = 9), and own fear of difficult situations emerging that may not be manageable (*n* = 1). Ten doctors also chose an open-ended answer category.

Below are the answers to this open-ended category of survey question #21 “What is the most challenging about suicide in patients with chronic skin conditions?” Multiple category assignment (Additional file 2: Appendix D1).

- ○ A patient should seek the conversation about it first (*n* = 2).
- ○ Most important is the proper treatment of the underlying disease and an open doctor-patient relationship (*n* = 2).
- ○ It is hard to recognize the suicidality in the patients (*n* = 2).
- ○ Lack of time in a contract practice (*n* = 1).
- ○ No challenge for a private practice (*n* = 1).
- ○ Patients’ motivation for a psychiatric treatment (*n* = 1).
- ○ Is barely accepted by patients (*n* = 1).

#### Part IV. Suicide prevention in dermatological practice

We were interested in the reaction of dermatologists to a specific suicide prevention plan.

Summarized below are participants’ responses to the open-ended question #22 “How would you evaluate the Picardi et al. (2013) prevention plan?” (*N* = 41). Multiple category assignment (Additional file 2: Appendix D2).

- ○ Positive reaction (*n* = 15)
  - ○ Such as good, helpful, ok.
- ○ Problems with implementation (problems that can occur during the implementation of the prevention):
  - ○ moderately practicable (*n* = 3);
  - ○ too time- or resource consuming (*n* = 3);
  - ○ problems with the data analysis (*n* = 3);
  - ○ talking about suicide can have an adverse effect (*n* = 2);
  - ○ difficulties in discussing this issue with the patients (*n* = 1);
  - ○ the patients’ poor acceptance of surveys (*n* = 1);
  - ○ Questionable cost-benefit ratio (*n* = 2).
- ○ Restriction in implementation (only parts of the prevention plan should/could be implemented):
  - ○ clinical dialogues are more appropriate than questionnaires (*n* = 2);
  - ○ only in case of a clinical indication (*n* = 2);
  - ○ only questionnaires, since clinical dialogues are too time-consuming (*n* = 1);
  - ○ a therapy must be done by a psychiatrist (*n* = 1).
- ○ Others (everything not falling into the other three categories):
  - ○ a similar prevention plan has already (partially) been implemented (*n* = 3);
    - ▪ For example, Dermatology Life Quality Index Questionnaire [43];
  - ○ not familiar with this prevention plan/no opinion (*n* = 4);
  - ○ this should result out of the doctor-patient relationship (*n* = 1).

The majority of dermatologists (*n* = 34) opined that clinical psychologists or psychotherapists could assist them in their job. Below we summarize the answers in response to the open-ended answer category of the question #23 “Could clinical psychologists or psychotherapists assist you in your work? If yes, how?” Multiple category assignment (Additional file 2: Appendix D3).

- ○ Expected help from the side of mental health professionals:
  - ○ by taking over the psychological care of patients (*n* = 10), such as
    - ▪ symptom-oriented dialogues, treatment of depressions, facilitation of the acceptance of the disease;
    - ▪ professionally identify the causes of psychosomatic disorders;
    - ▪ promote a better understanding of patients’ individual needs.
  - ○ by developing questionnaires, brochures, websites, hotlines, support groups, etc. (*n* = 5);
  - ○ as a member of a team, especially in a hospital (*n* = 6);
  - ○ availability in a case of need (*n* = 4);
  - ○ low-threshold psychological treatment (*n* = 2);
  - ○ exchange of experience, quick appointments on both sides (*n* = 1);
  - ○ in specialized outpatient clinics or private practice (*n* = 1);
  - ○ interdisciplinary trainings (*n* = 1);
  - ○ doctor’s referral (*n* = 1).
- Problems with implementation:
  - ○ the difficulty with health insurance reimbursement, especially in contract physician practices (*n* = 3);
  - ○ these skin conditions do not really belong to the group of psychosomatic skin disorders in terms of pathogenesis or therapy (*n* = 1).
- ○ Restrictions in implementation: as an additional treatment place that runs independently from a dermatologist practice (*n* = 2).
- ○ Others: no good experiences with psychologists (*n* = 1).

In response to the question of whether dermatologists think that they would profit from more suicide-related training programs in their job, 27 answered “yes”, whereas 15 with “no”.

### Tests of the research hypotheses

#### Statistical analysis was performed using the SPSS 26 software

##### Hypothesis 1

**Hypothesis 1a: private specialists have fewer clients than contract physicians**

Following ANOVA-assumptions were not met: the significant test of normality for the group of private specialists (Shapiro-Wilk Test, W (26) = .79, *p* < .01) and the significant test of homogeneity of variances (Levene’s Test, F (2, 42) = 5.03, *p* < .05).

Descriptive data analysis was consistent with this hypothesis:

$$ Md{n}_{contract}=250> Md{n}_{\mathrm{contract}/\mathrm{private}}=155> Md{n}_{private}=36 $$

A Jonckheere-Terpstra test was significant (J-T (3) = 72, *z* = − 4.85, two-tailed *p* < .01; with a large effect size of *d* = 2.09 between the extreme groups), indicating a decrease in patient contacts across these groups.

**Hypothesis 1b: private specialists on average spend more time with their patients than contract physicians do**

Following ANOVA-assumptions were not met: the significant test of normality for the group of contract physicians (Shapiro-Wilk Test, W (11) = .83, *p* < .021) and private specialists (Shapiro-Wilk Test, W (26) = .82, *p* < .01); and the significant test of homogeneity of variances (Levene’s Test, F (2, 42) = 11.01, *p* < .01).

Descriptive data analysis was consistent with this hypothesis:

$$ Md{n}_{contract}=10< Md{n}_{\mathrm{contract}/\mathrm{private}}=15< Md{n}_{private}=30 $$

The Jonckheere-Terpstra test was significant (J-T (3) = 538.5, *z* = 5.59, two-tailed *p* < .01, with a large effect size of *d* = 3.02), indicating an increase in the duration of doctor’s consultations across these groups.

**Hypothesis 1c: private specialists more often than contract physicians tell their patients that additional psychological, psychotherapeutic, or psychiatric treatments could be helpful**

Following ANOVA-assumptions were not met: the significant test of normality for the group of contract physicians (Shapiro-Wilk Test, W (11) = .72, *p* < .01) and private specialists (Shapiro-Wilk Test, W (26) = .70, *p* < .01).

Descriptive data analysis was not consistent with this hypothesis:

$$ Md{n}_{contract}=1< Md{n}_{\mathrm{contract}/\mathrm{private}}=2\nleqq Md{n}_{private}=1 $$

$$ {M}_{contract}=1.09<{M}_{\mathrm{contract}/\mathrm{private}}=1.75\nleqq {M}_{private}=1.46 $$

The Jonckheere-Terpstra test was not significant (J-T (3) = 332, *z* = 1.04, two-tailed *p* = .30), thus indicating that there nominally was no significant difference between these ordered groups. The Kruskal-Wallis *H* test was also not significant (χ^2^(2) = 5.04, *p* = .08, with mean ranks 17.09 in the group of contract physicians, 28.75 in the group of contract/private dermatologists, and 23.73 in the group of private specialists). These results indicate that there were no between-group differences in telling patients that additional psychological, psychotherapeutic, or psychiatric treatments could be helpful.

**Hypothesis 1d: private specialists more often than contract physicians ask their patients about their emotional state than contract physicians do**

Following ANOVA-assumptions were not met: the significant test of normality for all groups (Shapiro-Wilk Test, *p* < .05).

Descriptive data analysis was not consistent with this hypothesis:

$$ Md{n}_{contract}=2\nleqq Md{n}_{\mathrm{contract}/\mathrm{private}}=1.50< Md{n}_{private}=2 $$

$$ {M}_{contract}=1.82\nleqq {M}_{\mathrm{contract}/\mathrm{private}}=1.75<{M}_{private}=2.19 $$

The Jonckheere-Terpstra test was one-sided significant (J-T (3) = 363.50, *z* = 1.77, two-tailed *p* = .077, with a medium effect size of *d* = 0.55). We divided the *p* value by two because we had an a priori directional hypothesis. Moreover, the positive sign of the standardized test statistic *z* rejected the H0 in favor of the H1. We further performed a Kruskal-Wallis *H* test. This test was not significant (χ^2^(2) = 3.70, *p* = .16, with mean ranks 19.50 in the group of contract physicians, 18.38 in the group of contract/private dermatologists, and 25.90 in the group of private specialists). We would cautiously interpret these results as suggestive for a significantly increasing trend across the groups. Clearly, fresh data from a larger sample are needed to substantiate this view.

##### Hypothesis 2

**Hypothesis 2a: female doctors more often than male doctors tell their patients that additional psychological, psychotherapeutic, or psychiatric treatments could be helpful**

Following t-test-assumptions were not met: the significant test of normality for both groups (Shapiro-Wilk Test, *p* < .01).

Descriptive data analysis was consistent with this hypothesis:

$$ {M}_{females}=1.56>{M}_{males}=1.33 $$

A Mann-Whitney *U* test was not significant (*U* = 210, two-tailed *p* = .38, mean ranks 24.83 for women and 21.78 for men). The results indicate that there was no difference between female and male doctors in telling their patients that additional psychological, psychotherapeutic or psychiatric treatments could be helpful in treating their skin conditions.

**Hypothesis 2b: female doctors more often than male doctors ask their patients about their emotional state**

Following t-test-assumptions were not met: the significant test of normality for both groups (Shapiro-Wilk Test, *p* < .01).

Descriptive data analysis was consistent with this hypothesis:

$$ {M}_{females}=2.39>{M}_{males}=1.78 $$

The Mann-Whitney *U* test was significant (*U* = 129.50, two-tailed *p* < .01, mean ranks 29.31 for women and 18.80 for men, large effect size: *d* = 0.85). The results indicate that female doctors significantly more often asked their patients about their emotional state than male doctors do.

##### Hypothesis 3

**Hypothesis 3a: length of work experience has an influence on how often the doctors tell their patients that additional psychological, psychotherapeutic, or psychiatric treatments could be helpful**

Following ANOVA-assumptions were not met: the significant test of normality for all groups (Shapiro-Wilk Test, p < .01), and the significant test of homogeneity of variances (Levene’s Test, F (3, 41) = 2.928, *p* < .05).

Descriptive data analysis was consistent with this hypothesis:

$$ {M}_1=1.43\ne {M}_2=1.54\ne {M}_3=1.58\ne {M}_4=0.83 $$

The Kruskal-Wallis *H* test was not significant (χ2(3) = 6.34, *p* = .096, with mean ranks of 23.50 for the group “10 years or less”, 24.88 for the group “11–20”, 25.75 for the group “21–30”, and 12.25 for the group “31 years or more”). The Jonckheere-Terpstra test also was not significant (J-T (4) = 323.5, *z* = − 1.09, two-tailed *p* = .28). The results indicate that length of work experience had no influence on how often doctors told their patients that additional psychological, psychotherapeutic, or psychiatric treatments could be helpful.

**Hypothesis 3b: length of work experience has an influence on how often doctors ask patients about their emotional state**

Following ANOVA-assumptions were not met: the significant test of normality for all groups (Shapiro-Wilk Test, *p* < .05).

Descriptive data analysis was consistent with this hypothesis:

$$ {M}_1=2.07\ne {M}_2=2.23\ne {M}_3=2.00\ne {M}_4=1.50 $$

The Kruskal-Wallis *H* test was not significant (χ2(3) = 4.67, *p* = .198, with mean ranks of 23.82 for the group “10 years or less”, 26.58 for the group “11–20”, 22.67 for the group “21–30”, and 14.00 for the group “31 years or more”). The Jonckheere-Terpstra test also was not significant (J-T (4) = 312, *z* = − 1.31, two-tailed *p* = .19). The results indicate that length of work experience had no influence on how often doctors asked their patients about their emotional state.

##### Hypothesis 4

**Hypothesis 4a: doctors with a background in psychology more often than those without one tell their patients that additional psychological, psychotherapeutic, or psychiatric treatments could be helpful**

Following t-test-assumptions were not met: the significant test of normality for both groups (Shapiro-Wilk Test, *p* < .05).

Descriptive data analysis was consistent with this hypothesis:

$$ {M}_{with}=1.91>{M}_{with out}=1.26 $$

The Mann-Whitney *U* test was significant (*U* = 94.5, two-tailed *p* < .05, mean ranks 31.41 for dermatologists with psychological background and 20.28 for those without; large effect size*: d* = 0.78). The results indicate that doctors with a psychological background more often told their patients that additional psychological, psychotherapeutic, or psychiatric treatments could be helpful in treating their skin conditions.

**Hypothesis 4b: doctors with a background in psychology more often than those without ask their patients about their emotional state**

Following t-test-assumptions were not met: the significant test of normality for both groups (Shapiro-Wilk Test, *p* < .05).

$$ {Mean\ ranks}_{with}>{Mean\ ranks}_{with out} $$

Descriptive data analysis was consistent with this hypothesis:

$$ {M}_{with}=2.36>{M}_{with out}=1.91 $$

The Mann-Whitney *U* test was significant (*U* = 122, one-tailed *p* < .05, mean ranks 28.91 for dermatologists with psychological background, whereas 21.09 for those without; medium effect size: *d* = 0.53). The results indicate that doctors with a psychological background asked their patients more often about their emotional state. For comparative overview, the results of all inferential statistical tests are displayed in Table 4.

**Table 4:** Results of Inferential Statistical Tests

Hyp.	Test statistic	Effect size		Conclusion
		Value	Magnitude^a^	
*H*_1a_	*J-T*(3) = 72^††^	*d* = 2.09	very large	Private specialists have fewer clients than contract physicians.
*H*_1b_	*J-T*(3) = 538.5^††^	*d* = 3.02	very large	Private specialists on average spend more time with their patients than contract physicians do.
*H*_1c_	*J-T*(3) = 332 χ^2^(2) = 5.04			No difference between contract and private doctors in telling their patients that additional psychological treatments could be helpful.
*H*_1d_	*J-T*(3) = 363.5* χ^2^(2) = 3.70	*d* = 0.55	medium	Private specialists ask their patients more often about their emotional state.
*H*_2a_	*U* = 210			No difference between male and female doctors in telling their patients that additional psychological treatments could be helpful.
*H*_2b_	*U* = 129.5^††^	*d* = 0.85	large	Female doctors ask patients more often about their emotional state.
*H*_3a_	χ^2^(3) = 6.34 *J-T*(4) = 323.5			Length of work experience does not have an influence on how often doctors tell their patients that additional psychological treatments could be helpful.
*H*_3b_	χ^2^(3) = 4.67 *J-T*(4) = 312			Length of work experience does not have an influence on how often doctors ask patients about their emotional state.
*H*_4a_	*U* = 94.5^†^	*d* = 0.78	large	Doctors with psychological background tell patients more often that additional psychological treatments could be helpful.
*H*_4b_	*U* = 122*	*d* = 0.53	medium	Doctors with psychological background ask patients more often about their emotional state.

*Note. Hyp.* Hypotheses; *J-T* Jonckheere-Terpstra test statistic; χ^2^ = Kruskal-Wallis *H* test statistic; *U* = Mann-Whitney *U* test statistic; *d* = Cohen’s *d* effect size (computed with an effect-size calculator for nonparametric statistical tests, (available online [44])

*p < 0.05 one-tailed; **p < 0.01 one-tailed; ^†^*p* < 0.05 two-tailed; ^††^*p* < 0.01 two-tailed

^a^Magnitude interpretation [45]: “small” *d ≈* 0.20; “medium” *d ≈* 0.50; “large” *d* ≈ 0.80

## Discussion

### Summary of findings

For the present survey, we developed a 10-min online questionnaire, the link to which was emailed to Austrian dermatologists. The completion rate of 75% was acceptable. Average completion rates in comparable studies ranged from 73 to 88% [46]. However, the response rate of about 10% in our study was comparatively low.

The sample for analysis comprised 45 participants (of which 18 were women). Length of work experience was similarly distributed across all the subgroups of dermatologists considered. Twenty-six doctors were working privately, eleven had signed a contract with an insurance company, and eight doctors were working both privately and with a contract (i.e., were contract/private dermatologists). Eleven dermatologists appeared to have an additional professional background in psychology, or visited suicide-related trainings in the past 5 years.

A recent review has shown that chronic skin disorders might be associated with increased suicide risk [2]. The aim of the current study was to estimate the rate of suicidal thoughts, suicide attempts, and completed suicides in patients with atopic dermatitis, psoriasis, or acne, as based on dermatologists’ reports. The ultimate goal of this account is to promote cooperation between Austrian dermatologists and mental health professionals in treating patients with these chronic skin conditions.

We were additionally aiming to accomplish three SUPRA goals. The goal–support and treatment–is that suicide risk groups are supported and treated as needed. The study results revealed a rather low rate of suicidal thoughts, suicide attempts, and suicides in patients with chronic skin conditions. More precisely, only three dermatologists reported more than five patients having committed suicide within the past year. In addition, seven dermatologists in the same period treated 1–10 patients who expressed suicidal thoughts. Concerning suicidal attempts, the doctors participating in this survey either had no such patients, or could not provide an estimate. These results are somewhat unexpected, as compared to the majority of extant empirical studies that showed higher rates of completed suicides, suicide attempts, and suicidal thoughts in patients with chronic skin conditions. For example, in one study from the UK, utilizing a similar design (*N* = 341), 86 dermatologists reported a total of 178 patients “who had attempted suicide associated with their skin disorder” [47]. Other studies [22] did not find significant associations between skin disorders and suicide-related behaviors. The major question would be: do *Austrian* patients with these skin conditions indeed not belong to the group with an increased suicide risk compared to the general population?

The next SUPRA goal is to spread the awareness and knowledge about suicide in the population. The majority of the sample knew that patients with atopic dermatitis, psoriasis, or acne have a higher suicide risk. They also knew that these patients more often suffer from suicidal thoughts, as compared to control groups. This information primarily came from training programs. For the participating dermatologists, most challenging about suicide was the lack of time and the lack of knowledge; for example, most of them never heard about the Austrian suicide prevention program SUPRA. The doctors in the sample reported asking their patients frequently about their emotional state. On the other hand, they rather infrequently noted that psychiatric or psychological treatments could be helpful in treating the skin conditions.

In case of facing patients expressing suicidal thoughts, dermatologists usually had a conversation about this, arranged a new appointment, or referred the patient to a specialist in psychiatry. All of these actions are intervention steps mentioned by [2] in their suicide prevention plan. Further research could fruitfully evaluate each of these steps separately and their combination in terms of their suicide-preventive potential. For example, should dermatologists more often prescribe licensed psychoactive drugs? Only one participant in the present sample would do it, when encountering a patient with suicidal ideation.

Even though the study results did not suggest a high suicide rate in patients with atopic dermatitis, psoriasis, or acne, the majority of dermatologists stated that they would like to participate in more training programs to counteract patient suicidality. They also expressed positive attitudes towards the suicide prevention plan proposed by [2] and towards cooperation with mental health professionals.

The inferential statistical analyses yielded a number of significant results. As expected, private specialists had fewer patients and spent more time with them than contract physicians did. More precisely, there was a significant trend across the three groups of contract physicians, contract/private dermatologists, and private specialists. Yet, these differences did not seem to influence treatment quality, in terms of telling patients that additional psychological treatments could be helpful, and asking them more often about their emotional state.

We also found evidence for gender differences, first of all, in the participation rate: consistent with the evidence from the review by [48], more men than women participated in the study, although this difference nominally was not significant. Second, there is evidence [35] that female physicians work more patient-centered. We observed similar effects: female dermatologists more often reported asking their patients about their emotional state. There were no gender differences with regards to telling patients that additional psychological treatments could be helpful. In contrast to other studies [36, 37], length of work experience had no influence on the quality of treatment.

A further key finding was that an additional psychological background of dermatologists did have an influence on the doctor-patient communication. Doctors with a professional background in psychology, or those who were participating in the suicide-related training programs in the past 5 years more often told their patients that additional psychological, psychotherapeutic, or psychiatric treatments could be helpful, and also more often asked their patients about their emotional state than doctors without a psychological background.

Finally, the SUPRA goal–quality assurance and expertise–stresses solid research concepts when it comes to suicide prevention. We strived to conduct a high-quality explorative study, and yet, there are some limitations in our design that need to be discussed in more details.

### Study limitations and direction for future research

#### Low response rate

The survey response rate (10%) was comparatively low, thus diminishing the representativeness of the study and the generalizability of the results.

In this section, we elaborate on possible reasons for this low response rate. First, we mostly contacted administrative staff and not the dermatologists. On the other hand, we spoke on the phone several times with dermatologists personally and they shown little interest in the survey or had no time to participate. For future research, we would advise finding a contact person within the population of interest, who would help in spreading the questionnaires. Second, for increasing the response rate some incentives would be helpful. Before sending the e-mail reminders we added, that for each participation we will donate one euro to the non-governmental organization Doctors Without Borders. Since the study was financed privately, we had limited resources and could not offer more.

Third, our questionnaire lasted 10 min. Future investigations may well benefit from reducing questionnaire length (perhaps down to 3–5 min), in order to possibly increase participation rates. Depending on the intended focus of research, different parts (Parts II, III, or IV) of the survey form we developed could be left out.

Forth, we opted for collecting data online, and this particular method of data acquisition may well be responsible for the low participation rate. Whilst some studies [49] indicated that web-based questionnaires usually achieve only slightly lower response rates, as compared to the traditional paper-pencil questionnaires, there is opposing meta-analytic evidence [50], attesting that web surveys tend to have noticeably lower response rates than mail surveys. The similar study by [39] achieved a much higher response rate sending their questionnaires by post (33%).

There might be additional factors that had an impact on the study response rate, such as differences in the underlying populations, time periods, or researcher characteristics. Sax et al. [48] concluded that response rates depend on the populations being studied and overall substantially declined over the past decades (from 60% in 1960 to merely 21% in 2003).

The time period is indeed one of the key variables that influence the response rate. A review on e-mail survey response rates has shown, that the early level of high responses (40–72%) in the 1980th - 1990th was probably due to the novelty of the e-mail surveys and this period is likely to be over [51]. Indeed, in the year 2000 the mean response rate was already 24% [51]. The author expects that “response rates to e-mail surveys will continue to decrease” [51].

The correctness of the reported response rate depends also on its computation. There are many ways of calculating the response rate. Clearly, it makes it challenging to compare the results between studies. Some researchers [50] stress the importance of the so-called minimum response rate (RR1). The minimum response rate is the number of complete surveys divided by the number of surveys (complete plus partial) plus the number of nonsurveys (refusals and breakoffs plus noncontacts plus others) plus all cases of unknown eligibility [50]. Based on this definition, our response rate (10%) is likely to be even overestimated.

The declining willingness to participate, also results from the demands of the present time. As [48] wrote, that we are nowadays “bombarded” with questionnaires. So it is not surprising, that doctors who are already struggling with a lack of time, were not willing to participate in our survey. The lack of time was a major problem when discussing what is the most challenging about suicide as well. So time deficit must be seen as the main obstacle of present life.

In our opinion, online questionnaires still have much more advantages compared to the paper-pencil version, therefore, should be viewed as the method of choice for future studies, taking all limitations into consideration. The study by [48] showed, that the highest response rate could be achieved, using “paper survey with the option to complete the survey online” (p. 423).

#### The third-person perspective

Several reasons appear tenable to account for the low rate of suicides, suicide attempts, and suicidal thoughts reported in this survey of dermatologists. First, the impact of these conditions on patient suicidality might indeed not be substantial, most likely due to the quality of care provided by Austrian dermatologists. New inquiries are needed to substantiate this assumption further, which should address suicidality from a first-person perspective (i.e., patients). Possible differences between first-person (patients) and third-person (dermatologists) perspectives conceivably would give rise to follow-up research, for example, why dermatologists underestimate their patients’ suicide risk. Second, dermatologists in Austria might be unaware of suicidal behaviors in their patients, including completed suicides, although the majority of dermatologists did not choose the option “I don’t know”, when they were asked how many patients committed suicide in the past year. The majority of doctors also stated that they would not have problems to recognize suicidality in their patients. From this constellation it can be concluded that dermatologists in this sample were confident that almost none of their patients indeed committed suicide.

Relatedly, patients with suicidal thoughts or suicide attempts might not seek medical help or might be referred to a hospital without contacting their resident dermatologist in the first place. On the other hand, atopic dermatitis, psoriasis, or acne are chronic and, in the majority of cases, not life-threatening skin disorders. For these reasons it can be expected that the long-term care of these disorders indeed is mostly done by resident dermatologists [52] who, over the years, see these patients multiple times. Close doctor-patient relationship are therefore expected, with doctors being able to recognize changes in the course of the disease and in the overall wellbeing of their patients. By doing so, dermatologists take on a gatekeeper role. Their tasks include to recognize acute crises of their patients and to undertake the necessary prevention and intervention steps, including patient referral to a hospital. Exactly these considerations led us to exclude solely employed dermatologists from the sampling frame and population of interest of the current study. However, future studies could profitably compare these two target groups (resident vs. solely employed dermatologists) in terms of suicidality characteristics of their patients.

#### Operationalization

During the data analysis, we created a new variable psychological background. Participants were referred to the group of dermatologists with a psychological background, when they had any degree in psychology or had certain psychological certificates but also if they attended suicide training programs within the past 5 years. The latter one can be a matter of discussion since the topic of these programs is suicide in particular and not psychology per se. In our study, we also did not distinguish between the participants, who chose several or just one answer category regarding psychological background.

We operationalized the quality of treatment in our study as, whether dermatologists ask their patients about their emotional state and, whether they tell their patients that psychological treatment could be helpful in treating their skin conditions. This operationalization does not take into account a direct treatment outcome, such as changes in clinical symptoms during the medical treatment. Whether psychological treatment does help and under which circumstances is still a matter of an ongoing research. So it must be stressed out, that the operationalization of this study addresses only a small part of the whole treatment program and should not be solely used to draw a conclusion about the quality of work of Austrian dermatologists.

#### Contract with an insurance company

In our sample, more private dermatologists than contract dermatologists participated. Given the fact, that contract dermatologists have much more patients and thus could provide the scientific community with more information about patients, it would be crucial for future studies to try to recruit more participants from this subgroup.

#### Questions

Question 3 (“With what insurance company have you signed a contract?”) can be shortened. As the data analysis has shown, we divided the sample into three groups: contract physicians, contract/private dermatologists, and private specialists. It is therefore not necessary to ask with what particular health insurance company the physician has a contract. It would be enough to know, if a dermatologist has signed a contract with any insurance company, works privately or both.

Additionally, the data analysis revealed an inconsistency in the answers to this question. Some dermatologists first claimed to work only privately, later on stated to have also contract patients and vice versa. To prevent such inconsistency in future studies, the question “With what insurance company have you signed a contract?” could be left out. Another option would be to use a filter variable in the online questionnaire, such as that those doctors who claim to work privately would be allowed to provide an estimate only about the number of private patient contacts. It is not clear, however, what the reason for the inconsistency in the answers was in the first place. We additionally checked the answers of these participants. Those for example, who did not mention in the third question, that they also have a contract with an insurance company, provided a large number of contract visits. So it cannot be argued, that these participants treat a very negligible number of “forgotten” patients. Other reasons might be that the participants did not read the question carefully, or were worried that the answer to this question would somehow reveal their identity. Since it was one of the main variables of interest, such inconsistency in answers had an impact on the data analysis.

Questions 14, 15, and 16 (“How many patients with atopic dermatitis, psoriasis, and acne did you have in the past year that (a) committed suicide, (b) attempted suicide, and (c) expressed suicidal thoughts?”, respectively) showed a floor effect: The majority of the participants answered that they had no patients who shown suicide-related behavior within the last 12 months. Here one could take a longer period of time, for example, “[ …] during your carrier as a resident dermatologist?”

Question 17 (“What are/would be your actions, when treating a patient at a higher suicide risk?”). This question could be split into two questions. The first question would be about actions that dermatologists usually take *when* this situation occurs. The second question would be about actions that dermatologists think they would take *if* such a situation occurs. Such way it would be possible to separate between the real behavior and the behavior a dermatologist thinks he or she would show.

The sub-question 19.1 (“What are the reasons why you don’t ask your patients about their emotional state?”), had to be answered only if a specific answer (“never”) was provided to a previous question 19 (“How often do you ask your patients about their emotional state?”). In our survey, it was almost never the case. Only one dermatologist said that s/he never asks his or her patients about their emotional state. Future studies could change the filter question, such as that the sub-question 19.1 should be answered when another specific answer to the Q. 19–for example, “rarely”–is provided as well.

In the Question 22, we asked dermatologists to rate the prevention plan of [2]. The described prevention has lost very important details, especially in the second part (suicide-centered dialogue). Therefore, the answers of dermatologists should be evaluated taking these limitations into account. Future studies could make a survey that would include only this one particular question (alongside with the socio-demographic questions). In this case, the prevention plan of [2] would need to be described in more details.

Open-ended questions, as well as questions with open-ended answer categories provide a lot of information. Although, the data analysis can be challenging. Based on our findings, future studies could a priori formulate the answer categories, which would not only ease the data analysis but also reduce the influence of raters, and therefore increase the objectivity of the results.

### Implications for practice

The present results also suggest that collaborations between dermatologists and mental health professionals (foremost, clinical and/or health psychologists and psychotherapists) seem not only promising, but rather desirable, at least according to the viewpoint of dermatologists in Austria. Such new and additional cooperations should, of course, not push the medical staff to their limits. The results also indicate that not all dermatologists are willing to work together with psychologists (“no” *n* = 8), or to participate in suicide-preventive training programs (“no” *n* = 15). This is understandable, since new cooperations would require additional time and costs. In addition, the topic of suicidality in dermatological practice at first glance might not appear as overly important. Nonetheless, prevention plans, such as the one advanced by [2], might benefit the effectiveness of existing treatment practices. It is emphasized that the majority of doctors in our sample expressed positive attitudes towards this prevention plan.

At the same time, the prevention plan of [2] frequently was perceived as too time- and resource consuming, especially for dermatologists having contracts with insurance companies, although [21] stated that their prevention plan would not lead to longer doctor visits. Nevertheless, it still appears to be challenging to address further critical issues besides main skin problems within the average 8 min which contract physicians can spend with their patients.

As [2] recommended, physicians could either administer questionnaires to their patients, or ask them directly about suicidality, or do both. Some time could be saved, when deciding for only one part of these basic preventive measures. However, which type of prevention is more suitable for dermatological practice certainly differs from one place to another. Therefore, the mixed and opposing reactions of dermatologists concerning these measures are unsurprising. Some see questionnaires as better suited for practice, because of permanent time pressure. However, how to score, analyze, and evaluate questionnaire responses cannot be taken for granted and anyway needs time as well. Other dermatologists opine that only suicide-centered dialogues add value information to diagnosis and therefore are optimal for practice. Then again, some dermatologists admit they do not exactly know how to address patients’ suicidality appropriately. Some assume that patients would be the first to seek conversation about it, or falsely believe that talking about suicide would even increase the risk of suicide.

Despite dialogues being more desirable, self-report questionnaires and surveys have advantages as well, such as standardization and straightforward data analysis. Several dermatologists mentioned that they already, at least partly, implemented the prevention plan outlined by [2]. To assess quality of life in patients with chronic skin conditions, they used the Dermatology Life Quality Index [43]. A decade of experience has shown that it is a simple, well validated tool and useful in dermatological sessions [53]. In addition, dialogues can be beneficial as a second step, i.e., in case of a clinical indication, such as when a questionnaire outcome is atypical, borderline, or suspicious.

Dermatologists and their patients would benefit from support from professionals with psychological background. This support must be provided without long bureaucratic hesitations or long waiting times for appointments. Our study clearly hints at tasks that mental health professionals (clinical and/or health psychologists or psychotherapists) could help with. Prior related research [20, 23] also highlights the importance of interdisciplinary teams in the treatment of skin conditions. Even though there is little doubt that such a multifactorial approach is indicated, it is less clear how it can be successfully implemented in everyday practice. In Austria, physicians usually have their own practice and mostly work independently from mental health professionals. Building a group practice requires time and is associated with costs. Even if dermatologists would refer some of their patients to psychotherapists, these patients would face additional costs, since not all insurance companies grant refunds for psychological or psychotherapeutic treatments.

In Austria, patients with psychological problems either can get help at a hospital or from self-employed psychotherapists. Psychological treatments provided by self-employed psychotherapists who are clinical and/or health psychologists usually are not fully reimbursed by insurance companies [54]. Therefore, patients with financial shortages most likely cannot afford long-term treatments. There are possibilities to get fully covered psychotherapeutic treatment (*Psychotherapie auf Krankenschein*), but this goes hand in hand with long waiting times and limited capacities [54]. The dermatologists participating in the present survey also stressed problems with health insurance refunds when addressing the possibility of working together with psychotherapists. It thus would be highly relevant to start developing new refund schemes, in order that ideally all patients in need could receive full treatment reimbursement.

Another problem, which mental health professionals can help to alleviate is patients’ poor acceptance of surveys. Psychological problems and mental disorders still are tabooed topics, and with the current account, we ultimately want to encourage patients with skin disorders to talk with their physicians about mental problems. In this context, one critical issue is that some patients with psychological problems just would not accept the need of psychiatric treatment [23, 55]. This could be an additional contributing factor for the rather low rate of suicide-related behaviors reported from dermatological practice, because patients may well think that such problems can or should not be discussed with their physician. Dermatologists and their patients would also profit from psychological brochures addressing the topic of suicidality in patients with skin disorders and providing contact information about psychological or psychiatric treatment possibilities in their vicinity. Whilst system-level changes require time, simple brochures, on the other hand, entail much less time or costs. Therefore, a first step that could be implemented directly is to develop and to distribute such brochures in dermatological ordinations.

## Conclusion

This survey-based research suggests that the prevalence of suicidal behaviors among dermatologists’ patients in Austria might be less pronounced than expected, as deduced from the recent international literature in psychodermatology and suicidology. However, dermatologists, especially in contract practices, are struggling with constant time pressure. As well, insufficient financial support of insurance funds could hinder the implementation of new suicide prevention and intervention programs. Further research is needed in order to specify a set of optimal conditions under which the prevention plan of [2], or similar approaches, could be successfully implemented. Without such system-level actions and changes, the role of psychological interventions and treatments in dermatology might remain underappreciated. Further implications for dermatological practice include: (a) to offer suicide-prevention training programs; (b) to distribute suicide-prevention brochures in dermatological practice; and (c) to address the suicidality problem in patients with chronic skin disorders from the first-person perspective (i.e., patients).

## Supplementary information

**Additional file 1.** SPSS syntax with the results and the code for data analysis.

**Additional file 2.**

## Data Availability

The study dataset, materials, and the SPSS-Syntax are accessible at https://osf.io/m78ra/ and are included in this published article [supplementary information files; Additional_file_1.pdf].

## Abbreviations

ÖÄK: Österreichische Ärztekammer
PSY: Diplomas in psychosocial (PSY-I), psychosomatic (PSY-II), and psychotherapeutic (PSY-III) medicine of the ÖÄK
SHI Fund: Social Health Insurance Fund
SUPRA: The Austrian suicide prevention programme
