# Supplementary material for: Dermatologists’ perceptions of suicidality in dermatological practice: a survey of prevalence estimates and attitudes in Austria
Source: BMC Dermatol. 2020 Sep 29;20:10. doi: 10.1186/s12895-020-00107-w (PMC7526254; doi:10.1186/s12895-020-00107-w)
Supplement: Supplementary file 1 — Additional file 1. SPSS syntax with the results and the code for data analysis. [file 12895_2020_107_MOESM1_ESM.pdf]

```
* Encoding: UTF-8.

* Um den Datensatz in SPSS zu laden, führen Sie diese Syntax bitte vollständig aus.

* - entweder (a) wählen Sie im Menü oben: "Ausführen", "Alles"

* - oder (b) markieren Sie die gesamte Syntax (Strg+A) und klicken Sie auf den Pfeil (Aktuellen Befehl ausführen)


DATA LIST FREE(TAB)
 /CASE (F8.0)
  SERIAL (A4)
  REF (A4)
  QUESTNNR (A4)
  MODE (A16)
  STARTED (DATETIME)
  SD01 (F3.0)
  SD02 (F3.0)
  SD03 (F8.0)
  SD03_01 (F1.0)
  SD03_02 (F1.0)
  SD03_03 (F1.0)
  SD03_04 (F1.0)
  SD03_05 (F1.0)
  SD03_06 (F1.0)
  SD03_07 (F1.0)
  SD03_08 (F1.0)
  SD03_08a (A64)
  SD04 (F3.0)
  SD05 (F3.0)
  SD06 (F8.0)
  SD06_01 (F1.0)
  SD06_02 (F1.0)
  SD06_03 (F1.0)
```

SD06_04 (F1.0)

SD06_04a (A128)

SD07 (F3.0)

SD08 (F3.0)

SD09 (F3.0)

SD09_01 (A64)

SD12 (F8.0)

SD12_01 (F1.0)

SD12_01a (F8.0)

SD12_02 (F1.0)

SD12_02a (F8.0)

SD15 (F8.0)

SD15_01 (F1.0)

SD15_01a (A8)

SD15_02 (F1.0)

SD15_02a (A16)

SD16 (F3.0)

R002 (F3.0)

R003 (F8.0)

R003_01 (F1.0)

R003_02 (F1.0)

R003_03 (F1.0)

R003_04 (F1.0)

R003_05 (F1.0)

R003_06 (F1.0)

R003_06a (A128)

R004 (F3.0)

R005 (F8.0)

R005_01 (F1.0)

R005_02 (F1.0)

R005_03 (F1.0)

R005_04 (F1.0)

R005_05 (F1.0)

R005_06 (F1.0)

R005_06a (A128)

R006 (F3.0)

R009 (F3.0)

R007 (F3.0)

R008 (F8.0)

R008_01 (F1.0)

R008_02 (F1.0)

R008_03 (F1.0)

R008_04 (F1.0)

R008_05 (F1.0)

R008_06 (F1.0)

R008_07 (F1.0)

R008_08 (F1.0)

R008_08a (A256)

PS02 (F3.0)

PS03 (F8.0)

PS03_01 (F1.0)

PS03_02 (F1.0)

PS03_03 (F1.0)

PS03_04 (F1.0)

PS03_05 (F1.0)

PS03_06 (F1.0)

PS04 (F3.0)

PS05 (F8.0)

PS05_01 (F1.0)

PS05_02 (F1.0)

PS05_03 (F1.0)

PS05_04 (F1.0)

PS05_05 (F1.0)

PS05_06 (F1.0)

PS06 (F3.0)

PS07 (F8.0)

PS07_01 (F1.0)

PS07_02 (F1.0)

PS07_03 (F1.0)

PS07_04 (F1.0)

PS07_05 (F1.0)

PS07_06 (F1.0)

PS07_06a (A256)

SP02_01 (A1024)

SP03 (F3.0)

SP03_02 (A512)

SP04 (F3.0)

TIME001 (F8.0)

TIME002 (F8.0)

TIME003 (F8.0)

TIME004 (F8.0)

TIME005 (F8.0)

TIME006 (F8.0)

TIME007 (F8.0)

TIME008 (F8.0)

TIME009 (F8.0)

TIME_SUM (F8.0)

MAILSENT (DATETIME)

LASTDATA (DATETIME)

FINISHED (F1.0)

Q_VIEWER (F1.0)

LASTPAGE (F8.0)

MAXPAGE (F8.0)

MISSING (F8.0)

MISSREL (F8.0)

TIME_RSI (F12.4)

DEG_TIME (F8.0).

BEGIN DATA

34 base interview 16-02-2018 13:38:23 1 2 3 1
2 2 1 2 1 1 1 2 1 1 2
1 1 1 2 2 1 654 1 2 64 1
1 2 978-987 1 3 1 1 1 1
2 1 1 1 1 1 1 1 2 1 1
1 2 3 3 2 1 2 1 1 1 1
1 2

föfyökläkföfköfköfkök
öökfyökfdökfdökgdökgf
ylgflgyljllfgf
fglgljkdklgkljkjlfd
ydljgllglj
kgfljkgfyljklfdlj
yfdlgfljkgfljkgfllf 4 1 1 1 1 2 1 1
4 1 1 1 1 2 1 1 2 1 1 2
1 1 1 1 o8 2 bjlk 2 19 10 19

9 17 9 6 6 13 108 16-02-2018 13:40:12 1 0
9 9 0 0 2.35 146

35 base interview 16-02-2018 13:56:12 2 5 1 2
1 1 1 1 1 1 1 2 2
2 1 1 Martin :-) 1 2 25
1 1 2 10-15 1 2 2
2
1 2 2 5 2 2 1 2 2 1
1 2 Test Martin 3
3 3 3 1 2
2 1 1 2 Martin Test

Ok, Zeitaufwand unbekannt 2 Test Martin 1 60 19 99 114 24
26 30 62 10 321 16-02-2018 14:03:36 1 0 9
9 0 0 1.08 15

36 base interview 17-02-2018 08:49:02 2 2 1 1
1 1 1 2 1 1 1 2 2
2 2 2 1 1 2
10 1 1 2 10 3 2
2
7 7 6 1 1 1 1 1 1 1 1
2 test 3 3
2 1 1 1 1 2
1 1 test 2 test 2 7 5 12 10 5
4 3 6 8 60

17-02-2018 08:50:02 1 0 9 9 0 0 2.68 256

37			base	interview		17-02-2018 14:54:25			1	2	2	2
	1	1	1	1	1	2	1		2	1	3	2
	2	2	1		2	1	2		1	2	10	1
		1	2	10	1		2	1	1	2	1	1
	1	1	1		1	1	2	1	1	1	1	1
		2	2	2	1	1	1	1	1	2	1	1
	1		4	1	1	1	1	1	2	1	4	1
	1	1	1	1	2	1	4	2	2	2	1	1
	1	1										
Test Nummer 4		1		2	30	28	57	75	30	13	26	18
	3	280		17-02-2018 14:59:05			1	0	9	9	0	0
	1.51	57										

39			base	interview		19-02-2018 13:55:13			1	3	1	1
	1	1	1	1	1	2	1		2	2		
					2	2	2		1	2	10	1
		1	2	10	1		3	2				
					2							
		5	5	5	1	1	1	1	1	1	2	1
	1		3								2	
							3	1	1	1	1	2
	1	1		test	2	test	2	6	7	10	9	2
	3	4	6	2	49							
19-02-2018 13:56:02			1	0	9	9	0	0	2.84	292		

41			base	interview		20-02-2018 14:46:51			1	5	1	1
	1	1	1	1	1	2	1		2	2		
					2	2	2		1	1		2
	37	1	1		2	30	3	1	1	1	1	1
	2	1	1		1	1	1	1	1	2	1	1
		1	1	1	2	2	1	1	2	1	1	1
	1		3								2	
							2	1	1	2	1	1
	1	1										
Problem mit der Auswertung/ den patienten darauf ansprechen für dermazologen schwierig,												1
		2	4	20	79	37	39	17	25	467	7	333
		20-02-2018 14:58:26			1	0	9	9	0	0	1.31	20

43			base	interview		22-02-2018 08:14:54			2	4	1	1
	1	1	1	1	1	2	1		2	2		
					2	2	2		1	1		2
	15	1	1		2	30	3	1	3	2	2	1
	2	1	1		1	3	2	2	1	2	1	1
		1	1	1	4	2	1	1	2	2	1	2
	1		3								2	

2 1 1 1 1 1
1 2

in der Privatordination ist dies keine nicht zu bewältigende Herausforderung setzen wir gelegentlich ein 2 durch Übernahme der psychologischen Betreuung/Management 1
6 28 36 44 64 17 159 113 73 359 22-02-2018 08:23:54 1 0

9 9 0 0 0.97 10

45 base interview 23-02-2018 09:18:26 2 5 1 1
1 1 1 1 1 2 1 2 2
2 2 2 1 1 2
8 1 1 2 30 3 1 1 1 1 1
2 1 1 1 1 1 1 1 2 1 1
1 1 1 3 2 1 1 2 1 2 1
1 3 2
1 1 2 1 1 1
1 1 gute Idee 2

im Spital bei onkologischen Patienten 1 13 19 65 303 64 15 323
281 2 537 23-02-2018 09:36:31 1 0 9 9 0
0 0.98 37

46 base interview 23-02-2018 12:48:46 1 5 1 1
1 1 1 1 1 2 1 2 1 1 1
1 1 2 Akupunktur 2 2 2 1 1
2 10 1 1 2 30 3 1 1 1 1
1 2 1 1 1 1 1 1 1 2 1
1 1 1 1 2 2 1 1 1 1 1
2 1 2 1
2 1 1 2 1
1 1 1 Gut 2

Teamarbeit 2 31 22 65 27 64 14 27 70 8 328
23-02-2018 12:54:14 1 0 9 9 0 0 1.2 15

47 base interview 23-02-2018 16:23:05 2 5 1 1
1 1 1 1 1 2 1 2 1 1 1
1 1 2 Notarzt
Angiologie 2 2 2 1 1
2 30 1 1 2 30 2 1 3 1
2 2 2 1 1 1 3 1 2 2 2
1 1 6 1 7 5 2 2 1 2 1
2 2 1 2
1 3 2 1 2
2 1 1 1

praktisches tool zur Wartezeitüberbrückung, aber wie mit dem Ergebnis schließlich umzugehen ist, ist zu hinterfragen. Bei DLQI Befragungen gehe ich auf das Thema der Suizid Gedanken u/o

Gefährdung ein und denke, daß dies ausreichend ist. Vorbeugend ist jedenfalls Empathie sicherlich sehr wichtig....

2

Umgang mit bzw Akzeptanz der Erkrankung fördern;
teilw Ursachen bei Psychosomatik professionell erfassen und bei Bedarf eine psychiatrischen Begutachtung / Therapie veranlassen
besseres Verständnis für die individuellen Bedürfnisse des Patienten aufbauen

1 6 25 55 33 54 9 31 323 9 545 23-
02-2018 16:32:11 1 0 9 9 0 0 1.25 16

48 base interview 25-02-2018 16:57:26 2 3 6 2
2 2 2 2 2 1 1 2 2
2 2 2 1 2 450 1
1 2 5 1 3 1 2 1 1 2
2 1 1 1 2 1 1 2 2 1 1
1 1 1 4 2 1 1 2 2 2 1
1 3 3
2 2 2 1 2 1
1 1

Das sollte sich im Rahmen der ärztlichen Beziehung zu PatientInnen ergeben 1 2
8 32 49 30 66 16 19 82 25 327 25-
02-2018 17:02:53 1 0 9 9 0 0 1.17 13

49 base interview 03-03-2018 12:50:54 1 3 6 2
2 2 2 2 2 1 1 2 1 1 1
1 1 2 akupunktur, orthomolekulare Medizin, angiolog. basisdiagnostik,
fortbildungsdiplom, sucht... 2 2 2 1 2 400 1
1 2 5-15 min 1 2 1 1 1 1 1
2 1 1 1

1 1 1 1 2 1 1 1 1 1 6 2
2 1 2 2 2 2 1 3
1
3 1 2 1 1 1 1 1 sehr gute Idee 2
fragebogen erarbeiten 1 4 28 141 51 40 18 63 56
8 409 03-03-2018 12:57:43 1 0 9 9 0 0
1.23 18

50 base interview 03-03-2018 19:03:46 2 2 7 2
2 2 2 2 2 2 1 2 1 1 1
1 1 2 Notarzt 2 2 2 2 2 150 2
10 2 2 5 2 20 2 1 2 1 1 1
1 2 2

Ein sehr guter Freund ist klinischer Psychologe, der in der akuten Krisenprävention mit akut Suizidalen arbeitet. 1 1 1 1 1 1 2 1 1
1 1 3 1 1 1 2 2 1 2 1
2 3

3 2 2 1 1 1 1 2

In der Kassensituation ist es kaum möglich, mit dem Dauerzeitmangel so eine Situation zuzulassen. Ich muss 50 Patienten pro 4 Stunden abarbeiten.

Ich arbeite vor allem mit Allergie Patienten. Es gibt DLQI Bögen für Urtikaria, Asthma, Neurodremitis, Rhinitis, Lebensmittelallergien etc., die im Alltag alle sehr hilfreich wären, aber alle vom Patienten sehr schlecht angenommen werden. Auch solche, die ich im Rahmen von Betreuung von Diplomanden auflege, werden kaum je ausgefüllt. Lange Fragebögen sind völlig unrealistisch in der Praxis. Nicht einmal unser im Qualitätsmanagement aufgelegter Fragebogen mit zwei Fragen wird mehr als zwei Mal pro Woche bei 750 Patienten ausgefüllt.

2 Teams, z. B. beim Neuodermitis Trainer. Die Schwierigkeit ist, die schlechte Verrechenbarkeit mit den Kassen. 1 42 31 94 37 199 24 297
560 54 402 03-03-2018 19:26:04 1 0 9 9 0
0 0.5 1

51 base interview 03-03-2018 19:44:55 2 2 1 1
1 1 1 1 1 2 1 2 2
2 2 2 1 1 2
8 1 1 2 30 3 1 1 1 1 1
2 1 1 2
1 1 1 1 2 1 1 1 1 1 1
1 3 2
3 1 1 1 1 2
1 1 hilfreich 2

Depressionen mit Patienten aufarbeiten 2 15 48 43 35 34 20 25
109 32 361 03-03-2018 19:50:56 1 0 9 9 0
0 1.04 10

52 base interview 03-03-2018 23:46:43 2 3 7 2
2 2 2 2 2 2 1 2 2
2 2 2 2 2 200 2
10 2 2 5 2 25 3 1 1 1 1 1
2 1 1 1 1 1 1 1 2 1 1
7 7 1 1 1 1 1 2 1 1 1
1 2 3
3 3 2 1 2 2
1 1

Nicht damit auseinandergesetzt 2 Unabhängig von der Dermatolog Behandlung 1 17
61 80 41 62 29 34 107 22 418 03-03-2018
23:54:16 1 0 9 9 0 0 0.78 0

55 base interview 04-03-2018 16:50:55 1 2 1 1
1 1 1 1 1 2 1 2 1 1 1
1 1 2 Klinischer prüfarzt, notarztdiplom 2 2 2
1 1 2 100 1 1 2 20 3 1
1 1 1 1 2 1 1 1 1 1 1

1 2 1 1 7 7 1 2 1 1 1
2 2 1 1 1 3
2 3 1
1

2 1 1 1 1 Gute Idee, gutes hilfstool 2 Broschüren,
Fortbildungen interdisziplinär 1 3 50 65 59 45 38 111 102
17 490 04-03-2018 16:59:05 1 0 9 9 0 0
1.01 14

56 base interview 05-03-2018 09:11:00 -9 -9 0 1
1 1 1 1 1 1 1 -9

3 33
36 05-03-2018 09:11:36 0 0 2
2 100 100 1.89 54

57 base interview 05-03-2018 11:40:04 1 2 1 1
1 1 1 1 1 1 2 angestellt 2 1 1
1 1 1 2 Fortbildungsdiplom 2 2 2 1
2 75 1 1 2 10-15 1 3 1 1
1 1 1 2 1 1 1 1 1 1 1
2 1 1 6 1 1 1 1 1 1 2
1 1 1 1 3
3 2 1 2
1 1

1 1 1 gut 1 1 5 47 192 46 58
25 61 72 20 409 05-03-2018 11:48:50 1 0 9
9 0 0 0.9 8

58 base interview 05-03-2018 14:06:51 2 4 1 1
1 1 1 1 1 2 1 1

11 27 8
46 05-03-2018 14:07:37 0 0 3
3 0 0 1.61 139

59 base interview 05-03-2018 14:08:16 2 4 1 1
1 1 1 1 1 2 1 2 2

					2	2	2		1	1		2
	10	1	1		2	20 Minuten		2	1	2	1	2
	1	2	1	1		1	2	1	2	1	2	1
	1		1	1	1	3	2	1	1	2	1	2
	1	1		3								2
								3	1	1	2	1
	1	1	1						2	15	68	41
	73	19										
54			272		05-03-2018 14:12:48			0	0	7	7	0
	0	1.31	33									
60			base	interview		05-03-2018 14:37:12			2	4	5	2
	2	2	2	1	2	1	1		2	2		
					2	2	2		2	2	50	2
	5	2	2	10	2	10	3	1	1	1	1	1
	2	1	1		1	1	1	1	1	2	1	1
		1	1	1	3	2	2	1	2	1	1	1
	1		3								3	
							2	1	2	1	1	1
	1	1		ok	2	Broschüren		1	3	21		
372	297	38	14	24	305	23	544		05-03-2018 14:55:29			1
	0	9	9	0	0	1.07	20					
61			base	interview		05-03-2018 20:55:24			2	2	1	1
	1	1	1	1	1	2	1		2	1	1	1
	1	1	2	Zertifikat ärztliche Wundbehandlung					2	2	2	
	1	1		2	20	1	1		2	15	3	1
	2	1	2	1	2	1	1		1	2	1	2
	1	2	1	1		1	7	1	6	2	2	1
	2	2	2	2	1		3					
			2								2	1
	1											
1	1	2	1	1								

Als allgemeine Maßnahme für alle Patienten mit den genannten Erkrankungen nicht sinnvoll. Wie alle anderen diagnostischen Maßnahmen, sollte auch diese nur gezielt und bei klinischem Verdacht/Bedarf eingesetzt werden. Fragebögen ersetzen nicht das Fragen!!!

2	Durch Einsatz bei Bedarf und Patientenwunsch.						1	3	26	77	44	58
	14	37	190	10	459		05-03-2018 21:03:03			1	0	9
	9	0	0	1.22	16							
62			base	interview		06-03-2018 11:51:06			1	2	1	1
	1	1	1	1	1	2	1		2	1	1	1
	1	1	2	notarztdiplom		2	2	2		1	1	
	2	40	1	1		2	30	3	2			
						2						
			1	7	1	2	1	1	1	2	1	1

2 1 2 1
2 2 1 2 1
2 1 1 schweirig in der Praxis 2

aktives Zugehen auf den Patienten , psychologisches gespräch mit dem Patienten 2 5
26 51 52 17 15 31 58 29 284 06-03-2018
11:55:50 1 0 9 9 0 0 1.42 25

64 base interview 06-03-2018 15:51:50 -9 -9 0 1
1 1 1 1 1 1 1 -9

11 5
16 06-03-2018 15:52:06 0 0 2
2 100 100 1.93 105

66 base interview 06-03-2018 19:05:41 2 2 1 1
1 1 1 1 1 2 1 2 2
2 2 2 1 1 2
270 1 1 2 15 3 1 1 1 1 1
2 1 1 1 1 1 1 1 2 1 1
1 1 1 3 2 1 1 1 1 2 2
1 3 1
2 1 1 1 1 1
1 2

eine ordentliche Behandlung der Grundkrankheit und eine offene Arzt-Patienten-Beziehung, um die Grundkrankheit unter Kontrolle zu bekommen, dann geht es dem Patienten viel besser

in der Praxis zu aufwändig, nur im Anlassfall sinnvoll 2 Erreichbarkeit im Bedarfsfall 1
8 18 72 29 41 23 161 768 22 355 06-
03-2018 19:24:43 1 0 9 9 0 0 0.9 7

67 base interview 06-03-2018 19:48:15 1 2 1 1
1 1 1 1 1 2 1 2 1 1 1
1 1 2 Umweltmedizin 2 2 2 1 1
2 150 1 1 2 20 3 1 1 1 1
1 2 1 1 1 1 1 1 1 2 1
1 1 1 1 3 2 1 1 2 1 2
1 1 3 2
2 2 1 2 2
1 1 1

fraglicher Aufwand-Nutzen-Relation, da in Klinik und Praxis bisher nie ein Suizid auftrat 1
1 7 31 95 42 49 29 36 107 17 413
06-03-2018 19:55:08 1 0 9 9 0 0 0.94 2

69 base interview 06-03-2018 22:08:58 2 3 1 1
1 1 1 1 1 2 1 2 2
2 2 2 1 1 2
30 1 1 2 30 3 1 1 1 1 1
1 1 2 n.a. 2
7 7 7 4 2 1 1 2 2 1 2
1 3 2
2 1 1 1 1 1
1 2

Bin offen dafür, stehe aber auf dem Standpunkt daß der Patient das Gespräch darüber suchen soll.
Klingt vernünftig 2

Akne ist gewiss keine, Psoriasis und Neurodermitis sind nur sehr bedingt psychosomatische Hautkrankheiten im Sinne der Pathogenese bzw. der Therapie
Bei hohem Leidensdruck bzw. Problemen der Akzeptanz einer chronischen Dermatose kann jedoch eine psychiatrische 7 psyhologische Intervention sehr hilfreich sein.

2 10 42 55 37 38 19 89 201 32 523 06-
03-2018 22:17:41 1 0 9 9 0 0 0.85 4

70 base interview 07-03-2018 14:47:31 1 3 1 1
1 1 1 1 1 2 1 2 1 1 1
1 1 2 Psychotherapeutin, Kurärztin 2 2 1 beides 2
2 0 2 30 1 1 2 30-50 3 1 2
1 2 1 1 2 1 1 3 2 2 1
1 2 1 1 1 2 3 2 2 1 2
1 1 1 1 1
1 1

1 1 1 1 1 1 2 Motivation des Patienten zum Psychiater
hilfreich 2 Als Team-Mitglied 1 17 34 101 30 83
18 261 54 12 386 07-03-2018 14:57:41 1 0 9
9 0 0 0.92 8

71 base interview 07-03-2018 15:37:16 2 3 6 2
2 2 2 2 2 1 1 2 2
2 2 2 1 2 300 1
1 2 3-5 1 3 1 1 1 1 1
2 1 1 1 1 1 1 1 2 1 1
1 7 1 3 2 1 1 2 2 1 1
1 3 3
2 2 2 2 1 1
1 1 Könnte sinnvoll sein 2

Übernahme der symptomorientierten Gespräche 1 16 23 232 41 55
22 31 99 25 387 07-03-2018 15:46:20 1 0 9
9 0 0 0.84 2

72 base interview 07-03-2018 19:04:57 2 3 7 2
2 2 2 2 2 2 1 2 2 0 1

1 1 1 2 1 2 1 2 250 1
1 2 13 1 3 1 1 1 1 1
2 1 1 1 1 1 1 1 2 1 1
1 1 1 3 2 1 1 2 1 2 1
1 2 1
3 1 1 2 1 1
1 1

Nicht bekannt, aber im Psoriasis Komorbiditätsfrsgebogen enthalten 2 Als zusätzliche Anlaufstelle ohne dass es mich 1 Minute mehr Zeit kostet. 1 3 30 98 49
62 20 18 127 21 428 07-03-2018 19:12:05 1 0
9 9 3 3 1.19 18

73 base interview 07-03-2018 22:32:40 2 3 6 2
2 2 2 2 2 1 1 2 1 2 2
2 1 1 2 2 2 1 2 500 1
1 2 4-15 1 3 2
2
1 1 1 4 2 2 1 2 1 2 1
1 3 2
2 1 2 1 1 1
1 1

Fragebogen kann ich mir schlecht vorstellen (aber Picardi kann ja gerne in eine typische Kassenordination kommen), die Masse der Patient*Innen leidet nicht so massiv unter den häufig diskreten Hautveränderungen (ist mir aber bekannt, dass Schweregrad und subj. Belastung nicht korrlieren.
Wenn muß/müßte ich dies direkt ansprechen.
Der letzte Patient mit Suizidgedanken ist wegen eines Heuschnupfen bei mir in Behandlung

2 niedrigschwelliger Zugang zu Therapeut*Innen 2 21 24 94 49 39
21 153 388 24 697 07-03-2018 22:46:13 1 0 9
9 0 0 0.71 2

75 base interview 08-03-2018 18:58:56 2 4 7 2
2 2 2 2 2 2 1 2 1 1 1
1 1 2 Diverse Seminare für Psychologie bei chron.Patienten 2 2
2 1 2 250 1 1 2 5 Min 1
3 2 2
1 1 1 3 2
2 1 2 1 1 1 1 3
3

4 1 1 1 1 1 1 2 Kann keine Gedanken lesen Eher nicht empfehlenswert. Man könnte damit auch "schlafende Hunde " wecken .
2 Leider bisher keine guten Erfahrungen mit Psychologen .Wäre oft sehr froh darüber
1 20 29 141 36

86 27 229 202 20 598 08-03-2018 19:12:07 1 0 9
9 0 0 0.63 1

79 base interview 09-03-2018 14:22:03 1 3 6 2
2 2 2 2 2 1 1 2 2
2 2 2 1 2 350 1
1 2 ca 10min 1 3 1 2 1 2
1 2 1 1 1 2 2 1 1 1 1
2 Pat oder Angehörige haben das während der Ordination schon geäußert. 1
1 2 3 2

1 1 2 1 2 1 1 3
2 3
1 2 1 1 1 1 1 Fragebogen ja! Ansprechen wäre bei den meisten mit einem nicht zu bewältigendem Zeitaufwand verbunden. 2

Natürlich wäre ein Team Psychotherapeut/Dermatologe ideal. Aber wer bezahlt den Psychotherapeuten? Bezahlen wollen die wenigsten selber. Das muss die Krankenkasse erledigen. Man zahlt ja so viel ein. 1 19 60 107 44 105 23

54 331 42 751 09-03-2018 14:35:08 1 0 9 9 0
0 0.57 0

80 base interview 09-03-2018 16:00:48 1 2 1 1
1 1 1 1 1 1 2 In einem Klinikum , Schwerpunkt kh tätig 1

182
37 70 117

09-03-2018 16:05:37 0 0 3 3 0 0 0.61 1

81 base interview 09-03-2018 16:05:38 1 2 2 1
1 1 1 1 1 2 2 In einem Klinikum tätig 2 1
2 2 2 1 1 2 2 2 2 2
200 2 20 2 2 15 2 25 2 1 2 1
2 1 2 1 1 1 2 1 2 1 2
1 1 1 1 1 3 1 2 1 2 2
1 1 1 1
1 2 2 2 1
1 2

1 1 Sehr gut 2 Gemeinsame Gespräche mit pat 1 2
26 37 21 36 11 16 38 9 196 09-03-2018 16:08:54 1 0 9 9 0 0 1.96 58

82 base interview 11-03-2018 19:49:35 2 5 1 1
1 1 1 1 1 2 1 2 2
2 2 2 1 1 2
140 1 1 2 12 3 1 3 2 1 2

2 1 1 1 1 1 1 1 2 1 1
1 1 1 4 2 1 1 2 2 2 1
1 3 2
3 2 2 2 1 1
1 1 Positiv 2

Broschüren zum Austeilen und online Seiten mit Informationen und Kontaktmöglichkeiten (Hotline)
1 3 20 49 107 50 52 64 109 11 365
11-03-2018 19:57:20 1 0 9 9 0 0 1.15 18

84 base interview 12-03-2018 09:58:41 2 5 1 1
1 1 1 1 1 2 1 2 1 2 2
2 1 1 2 1 1 AG Psychodermatologie der ÖGDV.
1 1 2 80 1 1 2 20 2 1
1 1 1 1 2 1 1 1 1 1 1
1 2 1 1 1 1 1 5 2 2 2
2 1 1 2 1 2
2 2 1
1 1 1

1 2 1 ist ok 2 bei Bedarf zur Verfügung stehen; 1 3
27 100 276 61 34 65 82 29 442 12-03-2018
10:09:59 1 0 9 9 0 0 0.95 14

87 base interview 12-03-2018 20:05:49 1 3 1 1
1 1 1 1 1 2 1 2 2
2 2 1 AG Psychodermatologie 1 1
2 80 1 1 2 15 min 3 2
2
1 1 1 1 1 1 1 1 1
1 2 1 2
2 3 1 1 1
2 1 1 1

Im Rahmen einer derm Begutachtung kann man natürlich die allgem Befindlichkeit erfragen, jedoch eine Therapieplanung sollte/muß durch den Psychiater erfolgen.
Psychologische Maßnahmen wären oft wünschenswert, nur lässt sich eben nicht alles in der Hautsprechstunde abdecken.

2

Wäre ein tolles Aufgabengebiet in Spezialambulanzen.
 In einer PrivatPraxis kann man natürlich auch eine Spazialisierung auf Psychosomatik machen,
in einer Kassenpraxis wird es wohl mit der Krankenkassenerstattung schwierig werden.

1 98 12 131 55 33 25 39 816 29 439 12-
03-2018 20:26:27 1 0 9 9 0 0 0.82 9

88 base interview 12-03-2018 20:05:49 1 3 1 1
1 1 1 1 1 2 1 1

36 42
78 12-03-2018 20:07:07 0 0 2
2 0 0 0.44 0

90 base interview 12-03-2018 21:25:28 2 3 1 1
1 1 1 1 1 2 1 2 1 1 1
2 1 1 2 2 1 ÖGDV 2 2 100 2
50 2 2 20 2 20 3 1 1 1 1 1
2 1 1 1 1 1 1 1 2 1 1
1 7 2 2 2 1 1 1 1 1 2
1 2 1
2 2 2 2 1 1
1 1 guteIdee ! 2

feedback,
niedrig schwellige Anlaufstellen für Patienten die Erstberatung durch Spezialisten
benötigen 2 6 28 119 43 37 12 21 663 16 387
12-03-2018 21:41:13 1 0 9 9 0 0 1.08 12

104 base interview 20-03-2018 09:44:24 1 5 1 1
1 1 1 1 1 2 1 2 1 1 1
1 1 2 akupunktur 2 2 2 1 1
2 80 1 1 2 30 2 1 1 1 1
1 2 1 1 1 1 1 1 1 2 1
1 1 1 1 3 2 1 1 2 1 2
1 1 3 1
2 1 1 1 1
2 1 1 gut 2

broschüren,selbsthilfegruppen 1 4 28 67 37 42 17 39 58
13 305 20-03-2018 09:49:29 1 0 9 9 0 0
1.29 15

106 base interview 20-03-2018 14:57:26 2 4 1 1
1 1 1 1 1 2 1 2 1 1 1
1 1 2 Notarzt 2 2 2 1 1 2
50 1 1 2 20 3 1 1 1 1 2
1 1 1 1 1 1 1 2 1 1 1
1 1 1 1 1 1 1 2 1 1 1
1 3 3
3 2 1 2 2 1
1 1 Aufwendig. 2

Bei psyhodomat Hautetkrankungdn 2 5 12 48 30 33 32 36
99 15 310 20-03-2018 15:02:36 1 0 9 9 0
0 1.34 20

107 base interview 20-03-2018 16:02:21 2 5 6 2
2 2 2 2 2 1 1 1

13 25
38 20-03-2018 16:02:59 0 0 2
2 0 0 0.89 1

108 base interview 20-03-2018 19:06:48 1 5 2 1
1 1 1 2 1 2 1 2 1 1 1
1 1 2 2 2 2 1 1 2
35 1 1 2 20-30 min 3

29 36 137
202 20-03-2018 19:10:10 0 0
3 3 7

6 0.53 0

109 base interview 20-03-2018 19:10:14 1 5 2 1
1 1 1 2 1 2 1 2 2
2 2 2 1 1 2
35 1 1 2 30 min 3 1 2 1 2 1
2 1 1 1 2 1 2 1 2 1 1
1 7 1 2 2 1 1 2 1 1 1
1 3 2
2 2 2 1 1 2
1 1

Bei stark auffälligen DLQI Fragebogen (wird routinemäßig erhoben/ dermatologischer Lebensqualitätsindex) Frage ich gezielt nach dem Einfluss der Dermatose auf das psychische Wohlbefinden und je nach Antwort konkret nach Suizidgedanken

2 Mitbehandlung von psychisch auffälligen hautkranken Patienten1 7 14 32
38 148 22 32 303 10 508 20-03-2018 19:20:20 1
0 9 9 0 0 1.19 18

110 base interview 21-03-2018 17:28:47 2 4 3 1
2 2 1 2 1 1 1 2 1 1 1
1 1 2 n.a. (EP) 2 2 2 1 2 20
1 1 2 10 1 2 1 1 1 2
1 1 1 1 2
6 1 1 1 1 1 1 2 1 1

1 1 4 1 1 1 2 1 1 1 2
2 1 1 1 2
1 1 1

n.a. (EP)
Der Fragebogen kam mit dem Post!!! 1 2 16 34 83
131 32 160 22 35 4 285 21-03-2018 17:37:25 1
0 9 9 0 0 1.32 32

111 base interview 22-03-2018 10:28:53 2 2 1 1
1 1 1 1 1 2 1 2 2
2 2 2 1 1 2
10 1 1 2 30 3 2
2
1 1 1 3 2 2 1 1 1 2 1
1 2 2
3 1 1 1 1 1
1 2

Suizidalität spezifisch zu erkennen ist im Allgemeinen schwierig Fragebögen sind in der Praxis ungeeignet. 2 Mit dem Patienten sprechen und Zeit verbringen 2 2 13
37 27 30 18 211 147 6 317 22-03-2018
10:37:04 1 0 9 9 0

0 1.6 46

112 base interview 22-03-2018 12:45:01 1 2 1 1
1 1 1 2 1 1 1 2 2
2 2 1 AG Psychodermatologie der ÖGDV
2 2 25 2 25 2 2 15 2 15 3 2
2
6 7 1 4 2 1 1
2 2 1 2 1 3
2 3 1
1 1 1 1 1 2

Wird vom Patienten nicht thematisiert Schwierig, da DermatologInnen mit den Antworten selbst sicher nicht umgehen können. 2 Broschüren 2 4 23 60 38 26
13 40 67 7 278 22-03-2018 12:49:39 1 0 9
9 0 0 1.51 26

113 base interview 22-03-2018 13:43:40 1 5 1 1
1 1 1 1 1 2 1 2 2
2 2 2 1 1 2
25 1 1 2 30 min 3 2
1 1 1 1 1 2 1 1
1 1 1 3 2 2 1 2 1 1 1
1 2 2
2 4 2 2 2 2
1 1 gute idee 2 kontakt 1 13 15 58

42	133	11	42	88	3	322		22-03-2018 13:50:25			1	0
	9	9	0	0	1.31	31						
114			base	interview		22-03-2018 14:35:57			2	3	1	1
	1	1	1	1	1	2	1		2	2		
					2	1	1		0	1		1
		0	1		1		3					
								11	45	64		
					120		22-03-2018 14:37:57			0	0	3
	3	25	24	0.87	3							
115			base	interview		22-03-2018 15:28:04			2	5	1	1
	1	1	1	1	1	2	1		2	1	1	1
	1	1	2	Akkupunktur		2	2	2		1	1	
	2	15	1	1		2	30 minuten		3	1	2	2
	1	1	2	1	1		1	2	2	1	1	2
	1	1		1	1	2	3	1	1	1	2	2
	1	2	1		2							
	2								2	1	1	1
	2	1	1	1								
sinnvoll	1		2	11	17	66	30	67	10	67	159	6
	433		22-03-2018 15:35:17			1	0	9	9	0	0	
	1.22	18										
116			base	interview		22-03-2018 16:44:13			1	5	6	2
	2	2	2	2	2	1	1		2	1	1	1
	1	1	2	Notarzt Substitution			2	2	2		1	2
	160	1		1	2	5-15 min		1		3	1	1
	1	1	1	2	1	1		1	1	1	1	1
	2	1	1		1	1	1	3	1	2	1	2
	2	1	1	1		3						
		2								3	2	2
	1	1	2									
1	1						6	23	86	32	67	17
	43			274		22-03-2018 16:48:48			0	0	7	7
	0	0	1.08	8								
119			base	interview		23-03-2018 08:41:09			1	2	1	1
	1	1	1	1	1	2	1			2		

7 23
30 23-03-2018 08:41:39 0 0 2
2 0 0 1.24 12

120 base interview 23-03-2018 13:25:17 2 5 1 1
1 1 1 1 1 2 1 1

6 23
29 23-03-2018 13:25:46 0 0 2
2 0 0 1.36 18

121 base interview 23-03-2018 13:26:02 2 5 1 1
1 1 1 1 1 2 1 2 2
2 2 2 1 1 2
50 1 1 2 30 3 1 1 1 1 1
2 1 1 1 3 2 1 1 2 2 1
1 1 2 5 2 2 1 2 1 1 2
2

Soweit möglich Paktfähigkeit feststellen oder bei konkreter Gefahr Polizei u NA informieren 3
3
2 2 1 2 2 1 1 1
mäßig praktikabel 1 1 2 7 50 29 135 12
27 45 41 263 23-03-2018 13:31:50 1 0 9 9
0 0 1.65 53

122 base interview 23-03-2018 14:05:01 1 2 1 1
1 1 1 1 1 2 1 2 1 1 1
1 1 2 Kurarzt, Arbeitsmedizin 2 2 1
Psychodermatologie ÖGDV 1 1 2 150 1 1
2 15 min 3 1 1 1 1 1 2 1 1
1 1 1 1 1 2 1 1 1 7 1
3 2 2 1 2 1 1 1 1 2

2 2 1 1
1 1 1 1 2 Ich versuche, die Haut und Befindlichkeit des Pat. zu verbessern, so dass er / sie sich wohl fühlt

Ich bin mir nicht sicher, ob so ein Fragebogen nicht sogar eine Negativspirale in Gang bringen könnte. 2 Guter Austausch, rasche Termine auf beiden Seiten 2 9 25 97
34 50 11 113 111 9 459 23-03-2018 14:12:40 1
0 9 9 0 0

1.06 8

123			base	interview		24-03-2018 21:23:34			1	5	1	1
	1	1	1	1	1	2	1		2	1	0	1
	1	1	1		2	2	2		1	1		2
	10	1	1		2	30min	3					
								98	38	131		
					179		24-03-2018 21:28:01			0	0	3
	3	7	7									
0.45	0											
124			base	interview		26-03-2018 09:58:53			1	2	1	1
	1	1	1	1	1	2	1		2	1	2	2
	2	1	1		2	2	1	G Psychodermatologie der ÖGDV				
und DDG		1	1		2	120	1	1		2	15	3
	1	4	2	2	1	2	2	1		1	4	2
	2	1	2	2	1		1	1	2	3	2	1
	1	2	1	2	1	1		2				
				1								2
1	2	1	1	1	1	1		nach einem vorhergehenden				
eingehenden Gespräch willkommen					2	überweisung		1	10	40	101	39
	1065	18	21	68	24	371		26-03-2018 10:21:59			1	0
	9	9	0	0	0.92	7						
126			base	interview		05-04-2018 06:26:54			1	2	5	2
	2	2	2	1	2	1	1		2	1	1	1
	1	1	2	Arbeitsmedizin		2	2	2		1	2	200
	1		1	2	10	1		3	2			
						2						
			1	1	1	3	2	2	1	1	2	1
	1	1		3								2
								2	1	2	1	1
	1	1	1						42	36	194	44
	43	28										
36			272		05-04-2018 06:33:57			0	0	7	7	0
	0	0.73	1									
127			base	interview		08-04-2018 10:38:00			2	3	6	2
	2	2	2	2	2	1	1		2	1	1	1
	1	1	2	a	2	2	2		1	2	200	1
		1	2	5	1		2	1	1	1	2	1
	1	1	1		1	1	1	2	1	1	1	1
		1	1	1	2	1	1	1	2	1	2	1
	1		2								3	

2 1 1 1 1 1
1 2

Wird von Pat kaum akzeptiert praxisfern 1 2 29 37 120 30
37 15 59 46 25 398 08-04-2018 10:44:38 1 0
9 9 0 0 1 12

128 base interview 21-04-2018 22:50:46 2 5 1 1
1 1 1 1 1 2 1 2 1 1 1
1 1 2 Substitutionstherapie 2 2 2 2 2
120 2 10 2 2 10 2 40 3 1 1 2
1 1 1 1 1 1 1 2 1 1 1
1 1 7 7 2 1 1 1 1 2 1
1 1 1 2
2 3 1 2 1
1 1 1

1 Gut 1 1 23 39 238 85 105 34 113
140 13 583 21-04-2018 23:03:56 1 0 9 9 0
0 0.56 0

129 base interview 24-04-2018 17:23:31 2 5 6 2
2 2 1 2 2 2 1 2 1 1 1
1 1 2 Notarzt, Ernährung, Umwelt, Palliativ, Geriatrie, Sonographie,
Angiolog. Basis = Diagnostik 2 2 2 2 2 300 2 20
2 2 5 2 20 3 1 2 1 1 1 2
1 2

Komorbidität ist bekannt 1 1 1 1 1 1 1 2
DIESE und vorherige Antwort zu den Suizidgedanken wurden ausgelassen. Der Fragebögen
kam mit Post. EP!!! 7 7 1 2 2 1 1 2 1 1
1 1 3 3
4 1 1 2 1
1 1 1

Wer hat die Zeit?
Wer kennt sich aus? 2 v.a. im Spital 1 10 23 107
73 102 51 16 29 3 381 24-04-2018 17:30:25 1
0 9 9 0 0 1.39 43

END DATA.

VARIABLE WIDTH SERIAL REF QUESTNNR MODE SD03_08a SD06_04a SD09_01 SD15_01a
SD15_02a R003_06a R005_06a R008_08a PS07_06a SP02_01 SP03_02 (8) STARTED
MAILSENT LASTDATA (20)
.

**** Variable und Value Labels
****

VARIABLE LABELS

CASE 'Interview-Nummer (fortlaufend)'

SERIAL 'Seriennummer (sofern verwendet)'

REF 'Referenz (sofern im Link angegeben)'

QUESTNNR 'Fragebogen, der im Interview verwendet wurde'

MODE 'Interview-Modus'

STARTED 'Zeitpunkt zu dem das Interview begonnen hat (Europe/Berlin)'

SD01 'Geschlecht'

SD02 'Berufserfahrung'

SD03 'Krankenkasse: Ausweichoption (negativ) oder Anzahl ausgewählter Optionen'

SD03_01 'Krankenkasse: GKK (Gebietskrankenkasse)'

SD03_02 'Krankenkasse: BVA (Versicherungsanstalt öffentlich Bediensteter)'

SD03_03 'Krankenkasse: VA (Versicherungsanstalt für Eisenbahnen und Bergbau)'

SD03_04 'Krankenkasse: SVA (Sozialversicherungsanstalt der gewerblichen Wirtschaft)'

SD03_05 'Krankenkasse: KFA (Krankenfürsorgeanstalt der Bediensteten der Stadt Wien)'

SD03_06 'Krankenkasse: SVB (Sozialversicherungsanstalt der Bauern)'

SD03_07 'Krankenkasse: Privat/Wahlarzt bzw. -ärztin'

SD03_08 'Krankenkasse: Andere, und zwar'

SD03_08a 'Krankenkasse: Andere, und zwar (offene Eingabe)'

SD04 'Klinische/Gesund. psy'

SD05 'ÖÄK'

SD06 'ÖÄK Auswahl: Ausweichoption (negativ) oder Anzahl ausgewählter Optionen'

SD06_01 'ÖÄK Auswahl: Psychosoziale Medizin'

SD06_02 'ÖÄK Auswahl: Psychosomatische Medizin'

SD06_03 'ÖÄK Auswahl: Psychotherapeutische Medizin'

SD06_04 'ÖÄK Auswahl: Andere, und zwar'

SD06_04a 'ÖÄK Auswahl: Andere, und zwar (offene Eingabe)'

SD07 'Psychologiestudium'

SD08 'Fort-kurse Suizid'

SD09 'AG Psychodermatologie'

SD09_01 'AG Psychodermatologie: Ja, und zwar'

SD12 'Wie viele Kontakte K+P: Ausweichoption (negativ) oder Anzahl ausgewählter Optionen'

SD12_01 'Wie viele Kontakte K+P: Als Kassenarzt bzw. Kassenärztin'

SD12_01a 'Wie viele Kontakte K+P: Als Kassenarzt bzw. Kassenärztin (offene Eingabe)'

SD12_02 'Wie viele Kontakte K+P: Als Privat/Wahlarzt bzw. Privat/Wahlärztin'

SD12_02a 'Wie viele Kontakte K+P: Als Privat/Wahlarzt bzw. Privat/Wahlärztin (offene Eingabe)'

SD15 'Wie viele Minute K+P: Ausweichoption (negativ) oder Anzahl ausgewählter Optionen'

SD15_01 'Wie viele Minute K+P: Als Kassenarzt bzw. Kassenärztin'

SD15_01a 'Wie viele Minute K+P: Als Kassenarzt bzw. Kassenärztin (offene Eingabe)'

SD15_02 'Wie viele Minute K+P: Als Privat/Wahlarzt bzw. Privat/Wahlärztin'

SD15_02a 'Wie viele Minute K+P: Als Privat/Wahlarzt bzw. Privat/Wahlärztin (offene Eingabe)'

SD16 'SUPRA'

R002 'Suizidrisiko'

R003 'Suizidrisiko WIE: Ausweichoption (negativ) oder Anzahl ausgewählter Optionen'

R003_01 'Suizidrisiko WIE: Aus Erfahrung'

R003_02 'Suizidrisiko WIE: Gezielte Recherche (Fachliteratur, Google Scholar, u.a.)'

R003_03 'Suizidrisiko WIE: Zufällig auf das Thema gestoßen'

R003_04 'Suizidrisiko WIE: Während einer Weiter-/Fortbildung bzw. Ausbildung'

R003_05 'Suizidrisiko WIE: Über KollegInnen (informeller Informationsaustausch)'

R003_06 'Suizidrisiko WIE: Eigene Antwort'

R003_06a 'Suizidrisiko WIE: Eigene Antwort (offene Eingabe)'

R004 'Suizidgedanken'

R005 'Suizidgedanken WIE: Ausweichoption (negativ) oder Anzahl ausgewählter Optionen'

R005_01 'Suizidgedanken WIE: Aus Erfahrung'

R005_02 'Suizidgedanken WIE: Gezielte Recherche (Fachliteratur, Google Scholar, u.a.)'

R005_03 'Suizidgedanken WIE: Zufällig auf das Thema gestoßen'

R005_04 'Suizidgedanken WIE: Während einer Weiter-/Fortbildung bzw. Ausbildung'

R005_05 'Suizidgedanken WIE: Über KollegInnen (informeller Informationsaustausch)'

R005_06 'Suizidgedanken WIE: Eigene Antwort'

R005_06a 'Suizidgedanken WIE: Eigene Antwort (offene Eingabe)'

R006 'Wie viele Suizide'

R009 'Wie viele Suizideversuche'

R007 'Wie viele Gedanken'

R008 'Wie verfahren: Ausweichoption (negativ) oder Anzahl ausgewählter Optionen'

R008_01 'Wie verfahren: Führe ein Gespräch darüber'

R008_02 'Wie verfahren: Verabrede einen konkreten nächsten Termin mit PatientIn'

R008_03 'Wie verfahren: Verschreibe Psychopharmaka'

R008_04 'Wie verfahren: Überweise an eine/n Facharzt bzw. Fachärztin für Psychiatrie'

R008_05 'Wie verfahren: Empfehle öffentliche Beratungsstellen für Suizidgefährdete'

R008_06 'Wie verfahren: Beziehe Angehörige mit ein'

R008_07 'Wie verfahren: Überweise in eine psychiatrische Ambulanz oder Klinik'

R008_08 'Wie verfahren: Eigene Antwort'

R008_08a 'Wie verfahren: Eigene Antwort (offene Eingabe)'

PS02 'Besprechen'

PS03 'Gründe/PPP Behandlung: Ausweichoption (negativ) oder Anzahl ausgewählter Optionen'

PS03_01 'Gründe/PPP Behandlung: Zeitmangel'

PS03_02 'Gründe/PPP Behandlung: Wissensmangel'

PS03_03 'Gründe/PPP Behandlung: Gehört nicht zu den Aufgaben der DermatologInnen'

PS03_04 'Gründe/PPP Behandlung: Probleme, Kontakte zu den PatientInnen aufzubauen'

PS03_05 'Gründe/PPP Behandlung: Eigene Angst vor dem Aufkommen schwieriger Situationen, die ggf. nicht zu bewältigen sind'

PS03_06 'Gründe/PPP Behandlung: Eigene Antwort'

PS04 'Befragen/Seele'

PS05 'Gründe/Seele: Ausweichoption (negativ) oder Anzahl ausgewählter Optionen'

PS05_01 'Gründe/Seele: Zeitmangel'

PS05_02 'Gründe/Seele: Wissensmangel'

PS05_03 'Gründe/Seele: Gehört nicht zu den Aufgaben der DermatologInnen'

PS05_04 'Gründe/Seele: Probleme, Kontakte zu den PatientInnen aufzubauen'

PS05_05 'Gründe/Seele: Eigene Angst vor dem Aufkommen schwieriger Situationen, die ggf. nicht zu bewältigen sind'

PS05_06 'Gründe/Seele: Eigene Antwort'

PS06 'Suizid erkennen'

PS07 'Herausforderung: Ausweichoption (negativ) oder Anzahl ausgewählter Optionen'

PS07_01 'Herausforderung: Zeitmangel'

PS07_02 'Herausforderung: Wissensmangel'

PS07_03 'Herausforderung: Gehört nicht zu den Aufgaben der DermatologInnen'

PS07_04 'Herausforderung: Probleme, Kontakte zu den PatientInnen aufzubauen'

PS07_05 'Herausforderung: Eigene Angst vor dem Aufkommen schwieriger Situationen, die ggf. nicht zu bewältigen sind'

PS07_06 'Herausforderung: Eigene Antwort'

PS07_06a 'Herausforderung: Eigene Antwort (offene Eingabe)'

SP02_01 'Bewertung Picardi: [01]'

SP03 'Hilfe Psychologe'

SP03_02 'Hilfe Psychologe: Ja, folgendermaßen'

SP04 'Schulungen'

TIME001 'Verweildauer Seite 1'

TIME002 'Verweildauer Seite 2'

TIME003 'Verweildauer Seite 3'

TIME004 'Verweildauer Seite 4'

TIME005 'Verweildauer Seite 5'

TIME006 'Verweildauer Seite 6'

TIME007 'Verweildauer Seite 7'

TIME008 'Verweildauer Seite 8'

TIME009 'Verweildauer Seite 9'

TIME_SUM 'Verweildauer gesamt (ohne Ausreißer)'

MAILSENT 'Versandzeitpunkt der Einladungsmail (nur für nicht-anonyme Adressaten)'

LASTDATA 'Zeitpunkt als der Datensatz das letzte mal geändert wurde'

FINISHED 'Wurde die Befragung abgeschlossen (letzte Seite erreicht)?'

Q_VIEWER 'Hat der Teilnehmer den Fragebogen nur angesehen, ohne die Pflichtfragen zu beantworten?'

LASTPAGE 'Seite, die der Teilnehmer zuletzt bearbeitet hat'

MAXPAGE 'Letzte Seite, die im Fragebogen bearbeitet wurde'

MISSING 'Anteil fehlender Antworten in Prozent'

MISSREL 'Anteil fehlender Antworten (gewichtet nach Relevanz)'

TIME_RSI 'Maluspunkte für schnelles Ausfüllen'

DEG_TIME 'Maluspunkte für schnelles Ausfüllen'

.

VALUE LABELS

/SD01 1 'weiblich' 2 'männlich' -9 'nicht beantwortet'

/SD02 5 '10 Jahre oder weniger' 2 '11-20' 3 '21-30' 4 '31 Jahre oder mehr'

-9 'nicht beantwortet'

/SD03_01 SD03_02 SD03_03 SD03_04 SD03_05 SD03_06 SD03_07 SD03_08 SD06_01

SD06_02 SD06_03 SD06_04 SD12_01 SD12_02 SD15_01 SD15_02 R003_01 R003_02

R003_03 R003_04 R003_05 R003_06 R005_01 R005_02 R005_03 R005_04 R005_05

R005_06 R008_01 R008_02 R008_03 R008_04 R008_05 R008_06 R008_07 R008_08

PS03_01 PS03_02 PS03_03 PS03_04 PS03_05 PS03_06 PS05_01 PS05_02 PS05_03

PS05_04 PS05_05 PS05_06 PS07_01 PS07_02 PS07_03 PS07_04 PS07_05 PS07_06

1 'nicht gewählt' 2 'ausgewählt'

/SD04 SD05 SD07 SD08 R002 R004 SP04 1 'Ja' 2 'Nein' -9 'nicht beantwortet'

/SD09 1 'Ja, und zwar' 2 'Nein' -9 'nicht beantwortet'

/SD16 1 'Ja, ich bin mit dem Programm sehr gut vertraut'

2 'Ja, ich habe etwas von diesem Programm gehört' 3 'Nein'

-9 'nicht beantwortet'

/R006 R009 1 '0 bzw. keine' 2 '1' 3 '2' 4 '3' 5 '4' 6 'mehr als 5'

7 'Ich weiß es nicht' -9 'nicht beantwortet'

/R007 1 '0 bzw. keine' 2 '1-10' 3 '11-20' 4 '21-30' 5 '31-40' 6 'mehr als 40'

7 'Ich kann es nicht einschätzen' -9 'nicht beantwortet'

/PS02 PS04 1 'Immer' 2 'Häufig' 3 'Selten' 4 'Nie' -9 'nicht beantwortet'

/PS06 1 'Nein' 2 'Eher nein' 3 'Eher ja' 4 'Ja' -9 'nicht beantwortet'

```
 /SP03 2 'Ja, folgendermaßen:' 1 'Nein' -9 'nicht beantwortet'
 /FINISHED 0 'abgebrochen' 1 'ausgefüllt'
 /Q_VIEWER 0 'Teilnehmer' 1 'Durchklicker'
.

MISSING VALUES
 SD01 SD02 SD04 SD05 SD07 SD08 SD09 SD16 R002 R004 R006 R009 R007 PS02 PS04
 PS06 SP03 SP04 (-8,-9)
 SD03_01 SD03_02 SD03_03 SD03_04 SD03_05 SD03_06 SD03_07 SD03_08 SD06_01
 SD06_02 SD06_03 SD06_04 SD12_01 SD12_02 SD15_01 SD15_02 R003_01 R003_02
 R003_03 R003_04 R003_05 R003_06 R005_01 R005_02 R005_03 R005_04 R005_05
 R005_06 R008_01 R008_02 R008_03 R008_04 R008_05 R008_06 R008_07 R008_08
 PS03_01 PS03_02 PS03_03 PS03_04 PS03_05 PS03_06 PS05_01 PS05_02 PS05_03
 PS05_04 PS05_05 PS05_06 PS07_01 PS07_02 PS07_03 PS07_04 PS07_05 PS07_06 (-8)
.

**** data cleaning
*************************************************************************************
***********
*delete test cases.
SELECT IF (CASE ne 34 AND CASE ne 35 AND CASE ne 36 AND CASE ne 37 AND CASE ne 39).
EXECUTE.

*response rate.
FREQUENCIES MAXPAGE.

*delete empty case 56,58,64,80,88,107,108,114,119,120,123(missing) 57 (angestellt).
SELECT IF (CASE ne 56 AND CASE ne 58 AND CASE ne 64 AND CASE ne 80 AND CASE ne 88 AND CASE
ne 107
    AND CASE ne 108 AND CASE ne 114 AND CASE ne 119 AND CASE ne 120 AND CASE ne 123 AND
CASE ne 57).
EXECUTE.
```

```
*2 survey came per post and were entered manually.

*to do the string shorter for future transformations.

DO IF CASE = 110.

compute SP02_01 = REPLACE("n.a. (EP)<br>Der Fragebogen kam mit dem Post!!!","n.a. (EP)<br>Der Fragebogen kam mit dem Post!!!","999").

END IF.

EXECUTE.


*transform missings in the string into 999.

DO IF CASE = 59 OR CASE = 116 OR CASE = 126.

compute SP02_01 = "999".

END IF.

EXECUTE.


missing values SP02_01("999").


*Changing values to missings (the questionnaire came per post and this Q was not answered).

DO IF CASE = 129.

   compute R004 = $sysmis.

   compute R005 = $sysmis.

   compute R005_01 = $sysmis.

   compute R005_02 = $sysmis.

   compute R005_03 = $sysmis.

   compute R005_04 = $sysmis.

   compute R005_05 = $sysmis.

   compute R005_06 = $sysmis.

END IF.

EXECUTE.


DO IF CASE = 129.
```

```
compute R005_06a = REPLACE("DIESE und vorherige Antwort zu den Suizidgedanken wurden ausgelassen. Der Fragebögen kam mit Post. EP!!!","DIESE und vorherige Antwort zu den Suizidgedanken wurden ausgelassen. Der Fragebögen kam mit Post. EP!!!","999").

END IF.

EXECUTE.


missing values R005_06a("999").


FREQUENCIES ALL.

*##################Part I. Socio-demographic data#############################

*gender Q.1 and nonresponse bias analysis (binomial test). p=0.52  sample (18 vs 27); population (236 vs 214).

FREQUENCIES SD01.

*if women group 1 (0.52), if men (0.48).

NPAR TESTS

/BINOMIAL (0.48)=SD01.


*Length of work experience (Q. 2).

FREQUENCIES SD02.


*Contract with an insurance company"1" (Q. 3). #SD03_01:GKK, _02:BVA; _03:VA; _04:SVA; _05:KFA; _06:SVB; _07:private; _08:other

#-->new variable contract 1:contract; 2:contract+private; 3:private.


*compute contract1.


DO IF ((SD03_01 = 2 OR SD03_02 = 2 OR SD03_03 = 2 OR SD03_04 = 2 OR SD03_05 = 2 OR SD03_06 = 2) AND (SD03_07 = 1 AND SD03_08 = 1)).

     compute contract1 = 1.

       ELSE IF ((SD03_01 = 2 OR SD03_02 = 2 OR SD03_03 = 2 OR SD03_04 = 2 OR SD03_05 = 2 OR SD03_06 = 2) AND (SD03_07 = 2 OR SD03_08 = 2)).

     compute contract1 = 2.

     ELSE.
```

```
    compute contract1 = 3.
END IF.
EXECUTE.

value labels contract1 1 'contract' 2 'contract+private' 3 'private'.
EXECUTE.

FREQUENCIES contract1.

*Psychological background: SD06_01:PSY-I, _02:PSY-II; _03:PSY-III; SD07:psychology; SD08:Suicide training; SD09:psychodermatology
* -->new variable psychological background 0:no; 1:yes;



DO IF (MISSING(SD06_01) AND MISSING(SD06_02) AND MISSING(SD06_03)).
      DO IF (SD07 = 1 OR SD08 = 1 OR SD09 = 1).
         compute PsychBack = 1.
      ELSE.
         compute PsychBack = 0.
      END IF.
   ELSE IF (SD07 = 1 OR SD08 = 1 OR SD09 = 1 OR SD06_01 = 2 OR SD06_02 = 2 OR SD06_03 = 2).
      compute PsychBack = 1.
   ELSE.
      compute PsychBack = 0.
END IF.
EXECUTE.

*include case 75 Diverse Seminare f?r Psychologie bei chron.Patienten.
DO IF CASE = 75 AND PsychBack = 0.
   compute PsychBack = 1.
END IF.
```

```
EXECUTE.

value labels PsychBack 0 "without psych. background" 1 'with psych. background'.
EXECUTE.

FREQUENCIES PsychBack.

*Datei Aufteilen. PsychBack vs without.
SORT CASES BY PsychBack.
SPLIT FILE BY PsychBack.
*gender SD01; SD02 length of work experience; SD16 SUPRA; R002 Suicide risk; R004 suicidal thoughts
R006 How many suicides; R009 suicide attempts; R007 suicidal thoughts; PS02 Do you tell psy treatment important;
PS04 do you ask about emo state; PS06 recognize suicide; SP03 help; SP04 training; contract1.
FREQUENCIES SD01 SD02 SD16 R002 R004 R006 R009 R007 PS02 PS04 PS06 SP03 SP04 contract1.

SPLIT FILE OFF.

*SUPRA.
FREQUENCIES SD16.

*The average number of patient contacts and duration of doctor visits (Qs. 10-11).
*Patient contacts data cleaning.
*SD12_01a contract #case70 "0"

DO IF CASE = 70 AND SD12_01a = 0.
   compute SD12_01a = $sysmis.
END IF.
EXECUTE.

*Contacts duration (cleaning)
```

```
#SD15_01a contract

10 #case49 5-15min; 4 #case71 3-5; 9.5 #case73 4-15; 5 #case75 5 Min; 10 #case79 ca 10min; 10 #case116 5-15 min.


DO IF CASE = 49 AND SD15_01a = "5-15 min".
   compute SD15_01a = "10".
END IF.
DO IF CASE = 71 AND SD15_01a = "3-5".
   compute SD15_01a = "4".
END IF.
DO IF CASE = 73 AND SD15_01a = "4-15".
   compute SD15_01a = "9,5".
END IF.
DO IF CASE = 75 AND SD15_01a = "5 Min".
   compute SD15_01a = "5".
END IF.
DO IF CASE = 79 AND SD15_01a = "ca 10min".
   compute SD15_01a = "10".
END IF.
DO IF CASE = 116 AND SD15_01a = "5-15 min".
   compute SD15_01a = "10".
END IF.
EXECUTE.
*change type of the variable to numeric SD15_01a.
alter type SD15_01a(f1) SD15_01a(f6.1).
*#SD15_02a private
20 #case59 20 Minuten; 40 #case70 30-50; 15 #case87 15 min; 30 #case109 30 min
30 #case113 30 min; 30 #case115 30 minuten; 15 #case122 15 min


DO IF CASE = 59 AND SD15_02a = "20 Minuten".
   compute SD15_02a = "20".
```

```
END IF.
DO IF CASE = 70 AND SD15_02a = "30-50".
   compute SD15_02a = "40".
END IF.
DO IF CASE = 87 AND SD15_02a = "15 min".
   compute SD15_02a = "15".
END IF.
DO IF CASE = 109 AND SD15_02a = "30 min".
   compute SD15_02a = "30".
END IF.
DO IF CASE = 113 AND SD15_02a = "30 min".
   compute SD15_02a = "30".
END IF.
DO IF CASE = 115 AND SD15_02a = "30 minuten".
   compute SD15_02a = "30".
END IF.
DO IF CASE = 122 AND SD15_02a = "15 min".
   compute SD15_02a = "15".
END IF.
EXECUTE.
*change type of the variable to numeric SD15_02a.
alter type SD15_02a(f1) SD15_02a(f6.1).


*contract with an insurance company2, (Questions 11 visits duration; Q. 10 would result in n=11; n=9; n=25)
#SD15_01a - duration of visits with contract patients #SD15_02a...with private patients
#-->new variable contract 1:contract; 2:contract+private; 3:private.
DO IF (NOT MISSING(SD15_01a) AND MISSING(SD15_02a)).
      compute contract2 = 1.
ELSE IF (MISSING(SD15_01a) AND NOT MISSING(SD15_02a)).
       compute contract2 = 3.
```

```
          ELSE.
           compute contract2 = 2.
END IF.
EXECUTE.

value labels contract2 1 'contract' 2 'contract+private' 3 'private'.
EXECUTE.
FREQUENCIES contract2.



*########################################
#Sum of patient contacts
#SD12_01a - number of contacts with contract patients + #SD12_02a ....with private patients
#-->new variable contacts_SUM.

DO IF (NOT MISSING(SD12_01a) AND MISSING(SD12_02a)).
        compute contacts_SUM = SD12_01a.
ELSE IF (MISSING(SD12_01a) AND NOT MISSING(SD12_02a)).
         compute contacts_SUM = SD12_02a.
          ELSE.
           compute contacts_SUM =SD12_01a + SD12_02a.
END IF.
EXECUTE.

*########################################
#(Mean of) time duration with patients
#SD15_01a - time duration with contract patients * #SD15_02a ....with private patients
#-->new variable contacts_tmean

DO IF (NOT MISSING(SD15_01a) AND MISSING(SD15_02a)).
        compute contacts_tmean = SD15_01a.
```

```
ELSE IF (MISSING(SD15_01a) AND NOT MISSING(SD15_02a)).
        compute contacts_tmean = SD15_02a.
         ELSE.
          compute contacts_tmean = MEAN (SD15_01a, SD15_02a).
END IF.
EXECUTE.

*#descriptives by a group.
SORT CASES BY contract2.
SPLIT FILE BY contract2.
DESCRIPTIVES contacts_SUM.
DESCRIPTIVES contacts_tmean.
SPLIT FILE OFF.

*#########Part II. Suicide risk in patients with chronic skin conditions. ############
R002 Do you know that these patients are at a higher suicide risk? Q12
R003_01 Where have you learned this info? Q. 12_1.
FREQUENCIES R002 R003_01 R003_02 R003_03 R003_04 R003_05.

*R004 Do you know that these patients are suffering from suicidal thoughts? Q.13
R005_01 Where have you learned this info?. Q.13_1.
FREQUENCIES R004 R005_01 R005_02 R005_03 R005_04 R005_05.

*R006 How many suicides? R009 #How many suicide attempts?  R007 #How many suicidal thoughts?
.
FREQUENCIES R006 R009 R007.

*R008 Intervention steps in case facings? .
FREQUENCIES R008_01 R008_02 R008_03 R008_04 R008_05 R008_06 R008_07.

*#################Part III. The interaction between the mind and the skin
############################
```

```
PS02 #How often do you tell your patients  that psychological...treatments could be helpful?

PS04 #How often do you aks your patients about their emotional state?

PS06 #Recognizing suicide (Q.20)?.

FREQUENCIES PS02 PS04 PS06.


*PS07 #Most challenging about suicide (Q. 21)?.

FREQUENCIES PS07_01 PS07_02 PS07_03 PS07_04 PS07_05.

FREQUENCIES PS07_06a.


*##################Part III. The interaction between the mind and the skin
############################

*Prevention plan of Picardi. Open-ended question.

FREQUENCIES SP02_01.


*SP03 #Do you wish for a cooperation with clinical psychologists?

*SP04 #Do you wish for more suicide-related training programs?.

FREQUENCIES SP03 SP04.

FREQUENCIES SP03_02.


*##################Statistical data analysis ############################.

*Transforming the variables, so that answer categories are in the right order

PS02 #How often do you tell your patients  that psychological...treatments could be helpful?

PS04 #How often do you aks your patients about their emotional state?.


RECODE PS02(1=3) (2=2) (3=1) (4=0) INTO PS02_umc.

RECODE PS04(1=3) (2=2) (3=1) (4=0) INTO PS04_umc.

EXECUTE.


value labels PS02_umc 0 'never' 1 'sometimes' 2 'often' 3 'on all ocasions'.

value labels PS04_umc 0 'never' 1 'sometimes' 2 'often' 3 'on all ocasions'.

EXECUTE.
```

```
*descriptive statistics BY group and test of the assumptions for parametric tests.
EXAMINE VARIABLES=PS02_umc PS04_umc contacts_SUM contacts_tmean BY contract2
/PLOT BOXPLOT NPPLOT
/COMPARE GROUPS
/STATISTICS DESCRIPTIVES
/CINTERVAL 95
/MISSING PAIRWISE /*IMPORTANT!
/NOTOTAL.

*ANOVA together with Levenes test.
ONEWAY PS02_umc PS04_umc contacts_SUM contacts_tmean BY contract2
/STATISTICS DESCRIPTIVES HOMOGENEITY
/MISSING ANALYSIS.

* --> non-parametric alternatives.
*Hypothesis 1a: Private specialists have fewer clients than contract physicians.
NPAR TESTS /J-T=contacts_SUM BY contract2 (1,3)
/METHOD=EXACT.

*Hypothesis 1b: Private specialists spend on average more time with their patients than contract physicians do.
NPAR TESTS /J-T=contacts_tmean BY contract2 (1,3)
/METHOD=EXACT.

*Hypothesis 1c: Private specialists tell their patients more often than contract physicians that an additional psychological, psychotherapeutic or psychiatric treatment could be helpful.
NPAR TESTS /J-T=PS02_umc BY contract2 (1,3)
/METHOD=EXACT.

NPAR TESTS /K-W=PS02_umc BY contract2 (1,3)
/METHOD=EXACT.
```

*Hypothesis 1d: Private specialists ask their patients more often about their emotional state than contract physicians do.

```
NPAR TESTS /J-T=PS04_umc BY contract2 (1,3)

/METHOD=EXACT.


NPAR TESTS /K-W=PS04_umc BY contract2 (1,3)

/METHOD=EXACT.
```

*Hypothesis 2a: Female doctors are telling their patients more often than male doctors that an additional psychological, psychotherapeutic or psychiatric treatment could be helpful.

*SD01 Gender.

*descriptive statistics BY group and test of the assumptions for parametric tests.

```
EXAMINE VARIABLES=PS02_umc PS04_umc BY SD01

/PLOT BOXPLOT NPPLOT

/COMPARE GROUPS

/STATISTICS DESCRIPTIVES

/CINTERVAL 95

/MISSING PAIRWISE /*IMPORTANT!

/NOTOTAL.
```

*ANOVA together with Levenes test (to check an assumption of the ttest).

```
ONEWAY PS02_umc PS04_umc BY SD01

/STATISTICS DESCRIPTIVES HOMOGENEITY

/MISSING ANALYSIS.
```

* --> non-parametric alternatives.

```
NPAR TESTS

    /M-W= PS02_umc BY SD01(1 2)

    /STATISTICS=DESCRIPTIVES

    /MISSING ANALYSIS.
```

*Hypothesis 2b: Female doctors are asking their patients more often about their emotional state than male doctors do.

```
NPAR TESTS
    /M-W= PS04_umc BY SD01(1 2)
    /STATISTICS=DESCRIPTIVES
    /MISSING ANALYSIS.
```

*Hypothesis 3a: The length of work experience has an influence on how often the doctors tell their patients that an additional psychological, psychotherapeutic or psychiatric treatment could be helpful.

*SD02 length of work experience.

```
RECODE SD02(5=1) (2=2) (3=3) (4=4) INTO SD02_umc.
EXECUTE.

value labels SD02_umc 1 '10 or less' 2 '11-20' 3 '21-30' 4 '30 or more'.
EXECUTE.
```

*descriptive statistics BY group and test of the assumptions for parametric tests.

```
EXAMINE VARIABLES=PS02_umc PS04_umc BY SD02_umc
/PLOT BOXPLOT NPPLOT
/COMPARE GROUPS
/STATISTICS DESCRIPTIVES
/CINTERVAL 95
/MISSING PAIRWISE /*IMPORTANT!
/NOTOTAL.
```

*ANOVA together with Levenes test.

```
ONEWAY PS02_umc PS04_umc BY SD02_umc
/STATISTICS DESCRIPTIVES HOMOGENEITY
/MISSING ANALYSIS.
```

* --> non-parametric alternatives.

NPAR TESTS /K-W=PS02_umc BY SD02_umc(1,4)

/METHOD=EXACT.

NPAR TESTS /J-T=PS02_umc BY SD02_umc(1,4)

/METHOD=EXACT.

*Hypothesis 3b: The length of work experience has an influence on how often the doctors ask their patients about their emotional state.

NPAR TESTS /K-W=PS04_umc BY SD02_umc(1,4)

/METHOD=EXACT.

NPAR TESTS /J-T=PS04_umc BY SD02_umc(1,4)

/METHOD=EXACT.

*Hypothesis 4a: Doctors with a psychological background tell their patients more often than doctors without a psychological background that an additional psychological, psychotherapeutic or psychiatric treatment could be helpful.

*descriptive statistics BY group and test of the assumptions for parametric tests.

EXAMINE VARIABLES=PS02_umc PS04_umc BY psychBack

/PLOT BOXPLOT NPPLOT

/COMPARE GROUPS

/STATISTICS DESCRIPTIVES

/CINTERVAL 95

/MISSING PAIRWISE /*IMPORTANT!

/NOTOTAL.

*ANOVA together with Levenes test (to check an assumption of the ttest).

ONEWAY PS02_umc PS04_umc BY psychBack

/STATISTICS DESCRIPTIVES HOMOGENEITY

/MISSING ANALYSIS.

* --> non-parametric alternatives.

NPAR TESTS /M-W=PS02_umc BY psychBack (0,1)

/METHOD=EXACT.

*Hypothesis 4b: Doctors with a psychological background are asking their patients more often about their emotional state than doctors without a psychological background do.

NPAR TESTS /M-W=PS04_umc BY psychBack (0,1)

/METHOD=EXACT.
