# Supplementary material for: Dermatologists’ perceptions of suicidality in dermatological practice: a survey of prevalence estimates and attitudes in Austria
Source: BMC Dermatol. 2020 Sep 29;20:10. doi: 10.1186/s12895-020-00107-w (PMC7526254; doi:10.1186/s12895-020-00107-w)
Supplement: Supplementary file 2 — Additional file 2. [file 12895_2020_107_MOESM2_ESM.docx]

Supplementary Material

# APPENDIX A

## English Translation (performed by Pronizius Ekaterina, MSc.)

**Gender**

- female
- male

**Length of work experience** *(in years)*

- 10 years or less
- 11-20
- 21-30
- 31 years or more

**With which insurance company you have contract?** *(Multiple answers are allowed!)*

- GKK (Gebietskrankenkasse or Regional Health Insurance Fund);
- BVA (Versicherungsanstalt öffentlich Bediensteter or Austrian Insurance Fund for Civil or Public Servants);
- VA (Versicherungsanstalt für Eisenbahnen und Bergbau or Insurance Institution for the Austrian Railways & Mining Industry);
- SVA (Sozialversicherungsanstalt der gewerblichen Wirtschaft or Austrian Social Insurance Authority for Business);
- KFA (KFA – Krankenfürsorgeanstalt der Bediensteten der Stadt Wien or Health Care Institution of the Municipal Civil Servants of the City of Vienna); and
- SVB (Sozialversicherungsanstalt der Bauern or Austrian Farmers' Social Security Authority).
- Private consultant/physician
- other: __________

**Do you have one or more ÖÄK diploma?** *(In case you are still undergoing the training and have not finished it yet, please still answer with “yes”)*

- yes 🡪 go to the question 4.1
- no 🡪 go to the question 5

1. **Which of the following ÖÄK diploma have you acquired?** *(Multiple answers are allowed!; In case you are still undergoing the training and have not finished it yet, please still tick the appropriate option)*

- psychosocial medicine
- psychosomatic medicine
- psychotherapeutic medicine
- other: __________

**Do you additionally have a degree in psychology?** *(In case you are still undergoing the training and have not finished it yet, please still answer with “yes”)*

yes

no

**Are you additionally a clinical psychologist / health psychologist?** *(In case you are still undergoing the training and have not finished it yet, please still answer with “yes”)*

yes

no

**Have you visited in the past five years (2013 - 2017) suicide training programs?**

yes

no

**Are you a member of a psychodermatology working unit** *(e.g., „ESDaP European Society for Dermatology and Psychiatry“ http://www.psychodermatology.net/ oder AG Psychodermatologie der ÖGDV)*

yes: __________

no

**Have you ever heard about the SUPRA prevention program of Austria?**

yes, I am very familiar with the program

yes, I have heard something about this program

no

**Please provide an estimation of the average amount of patient visits a week** *(e.g. If a patient comes to you two times in one week, please count this as 2 contacts. Please enter a number - a rough estimate is enough).*

as a contract physician: __________

as a private specialist: __________

**Please provide an estimation of the average duration of your patient consultations** *(Certainly, the treatment time might vary depending on the symptoms. You can specify a time interval).*

as a contract physician: __________

as a private specialist: __________

**Part II**

**Suicide risk in patients with chronic skin conditions**

**Do you know that patients with atopic dermatitis, psoriasis, and acne are at a higher suicide risk?** ^1-4,8,9^

- yes 🡪 go to the question 12.1
- no 🡪 go to the question 13
  1. **Where have you learned that these patients are at a higher suicide risk?** *(Multiple answers are allowed!)*
     - from experience
     - deliberate research (scientific literature, Google Scholar etc.)
     - accidentally discovered
     - training programs
     - from colleagues (informal)
     - own answer: __________

**Do you know that patients with atopic dermatitis, psoriasis, and acne are suffering from suicidal thoughts more often than a control group?** ^1-4,8,9^

- yes 🡪 go to the question 13.1
- no 🡪 go to the question 14

13.1 **Where have you learned that these patients are suffering from suicidal thoughts more often than a control group?** *(Multiple answers are allowed!)*

- - - from experience
    - deliberate research (scientific literature, Google Scholar etc.)
    - accidentally discovered
    - training programs
    - from colleagues (informal)
    - own answer: __________

**How many of your patients with atopic dermatitis, psoriasis, and acne have committed suicide within the past 12 months (3/2017-2/2018)?**

0 / none

1

2

3

4

more than 5

I don’t know

**How many of your patients with atopic dermatitis, psoriasis, and acne have tried to commit suicide within the past 12 months (3/2017-2/2018)?**

0 / none

1

2

3

4

more than 5

I don’t know

**How many of your patients with atopic dermatitis, psoriasis, and acne were expressing suicidal thoughts within the past 12 months (3/2017-2/2018)?**

0 / none

1-10

11-20

21-30

31-40

more than 40

I can’t provide an estimation

**What are/would be your intervention steps in case of facing acutely suicidal patients with atopic dermatitis, psoriasis, and acne?**  *(Multiple answers are allowed!)*

having a conversation about it

arranging a new appointment with the patient

prescribing psychiatric medications

referring to a specialist in the psychiatry

recommending suicide counseling centers

involving relatives

referring to a psychiatric ambulance or a clinic

own answer: __________

**Part III**

**The interaction between the mind and the skin**

**Do you tell your patients with atopic dermatitis, psoriasis, and acne that psychological, psychotherapeutic or psychiatric treatments could be helpful in treating their skin conditions?** ^5-7^

on all occasions 🡪 go to the question 19

frequently 🡪 go to the question 19

rarely 🡪 go to the question 19

never 🡪 go to the question 18.1

18.1 **What are the reasons that you DON’T tell your patients with atopic dermatitis, psoriasis, and acne that psychological, psychotherapeutic or psychiatric treatments could be helpful in treating their skin conditions?** *(Multiple answers are allowed!)*

- - - lack of time
    - lack of knowledge
    - suicide-related subjects are not part of their job
    - problems to establish a contact with a patient
    - own fear of the emergence of difficult situations that may not be manageable
    - own answer: __________

**How often do you ask your patients with atopic dermatitis, psoriasis, and acne about their emotional state?**

on all occasions 🡪 go to the question 20

frequently 🡪 go to the question 20

rarely 🡪 go to the question 20

never 🡪 go to the question 19.1

19.1 **What are the reasons that you DON’T ask your patients with atopic dermatitis, psoriasis, and acne about their emotional state?** *(Multiple answers are allowed!)*

- - - lack of time
    - lack of knowledge
    - suicide-related subjects are not part of their job
    - problems to establish a contact with a patient
    - own fear of the emergence of difficult situations that may not be manageable
    - own answer: __________

**Are you/would you have troubles in recognizing suicidal intentions (suicidal thoughts, suicide plans, or suicidal actions) in your patients with atopic dermatitis, psoriasis, and acne?**

- - - no
    - rather no
    - rather yes
    - yes

**What is the most challenging for you regarding suicide in patients with atopic dermatitis, psoriasis, and acne?** *(Multiple answers are allowed!)*

- - - lack of time
    - lack of knowledge
    - suicide-related subjects are not part of their job
    - problems to establish a contact with a patient
    - own fear of the emergence of difficult situations that may not be manageable
    - own answer: __________

**Part IV**

**Suicide prevention**

Picardi et al. (2006; 2013)^8,9^ suggest that dermatologists could help to prevent suicide in patients with chronic skin conditions by providing them with brief depression questionnaires (such as Patient Health Questionnaire PHQ). If such depression screening is not possible, the physician could directly inquire about it.

**How would you evaluate the Picardi et al.´s (2013) prevention plan?** ^8,9^

- own answer: __________

**Could clinical psychologists or psychotherapists assist you in your work? If yes, how?** *(e.g., by developing brochures or by forming teams (psychologist-dermatologist) in the treatment of psychosomatic skin disorders)?*

- no
- yes, as follows: __________

**Would you profit in your job from more suicide-related training programs?**

- yes
- no

**Thank you!**

**References**

**1.** Dalgard, F. J., Gieler, U., Tomas-Aragones, L., Lien, L., Poot, F., Jemec, G. B. E. Misery, L., ... Kupfer, J. (2015). The psychological burden of skin diseases: a cross-sectional multicenter study among dermatological out-patients in 13 European countries. *Journal of Investigative Dermatology, 135,* 984-991.

**2.** Dieris-Hirche, J., Gieler, U., Kupfer, J. P., & Milch, W. E. (2009). Suicidal ideation, anxiety and depression in adult patients with atopic dermatitis. *Der Hautarzt, 60*, 641-646.

**3.** Noh, H.-M., Cho, J. J., Park, Y. S., & Kim, J.-H. (2016). The relationship between suicidal behaviors and atopic dermatitis in Korean adolescents. *Journal of Health Psychology, 21*, 2183-2194.

**4.** Harth, W., Hillert, A., Hermes, B., Seikowski, K., Niemeier, V., & Freudenmann, R. W. (2008). Suizidalität in der Dermatologie. *Der Hautarzt, 59,* 289-296.

**5.** Mitschenko, A. V., Lwow, A. N., Kupfer, J., Niemeier, V., & Gieler, U. (2008). Neurodermitis und Stress. *Der Hautarzt, 59,* 314-318.

**6.** Ghosh, S., Behere, R. V., Sharma, P., & Sreejayan, K. (2013). Psychiatric evaluation in dermatology: An overview. *Indian Journal of Dermatology. 58*, 39-43.

**7.** Gieler, U., & Gröne, D. H. (2014, Oktober), Haut und Psyche: Ohne Psychodermatologie keine Practiceführung. *Ästhetische Dermatologie, 5.*

**8.** Picardi, A., Mazzotti, E., & Pasquini, P. (2006). Prevalence and correlates of suicidal ideation among patients with skin disease. *Journal of the American Academy of Dermatology, 54*, 420-426.

**9.** Picardi, A., Lega, I., & Tarolla, E. (2013). Suicide risk in skin disorders. *Clinics in Dermatology, 31,* 47-56.

# APPENDIX B

## The List of Internet Databases Provided by the Medical Associations of Each Federal State

- Vienna: <http://www.praxisplan.at/>;
- Carinthia: [http://www.aekktn.at/web/arztekammer-fur-karnten/arztsuche#](http://www.aekktn.at/web/arztekammer-fur-karnten/arztsuche);
- Burgenland: <http://www.aekbgld.at/web/arztekammer-fur-burgenland/arztsuche>;
- Lower Austria: <https://sso.arztnoe.at/arztsuche/search.jsf>;
- Upper Austria: <https://app.wigeogis.com/aekooe/>;
- Salzburg: <http://www.aeksbg.at/web/arztekammer-fur-salzburg/arztsuche>;
- Styria: <https://www.aekstmk.or.at/46>;
- Tirol: <http://www.aektirol.at/arztsuche/>;
- Vorarlberg: <https://www.medicus-online.at//aek/dist/medicus.html#sucheAllgemein>.

## Number of Search Results Separately for Each Federal State

- Vienna: 261;
- Carinthia: 31;
- Burgenland: 19;
- Lower Austria: 112;
- Upper Austria: 95;
- Salzburg: 49;
- Styria: 75;
- Tirol: 50;
- Vorarlberg: 24.

Total of 716 search results.

## Sending an Invitation: Separate Results for Each Federal State

1. The first data collection wave (total of 438 delivered e-mails + 12 by post):

- Vienna: 155;
- Carinthia: 21 (excl. 3 e-mails, that could not be delivered);
- Burgenland: 8;
- Lower Austria: 64 (excl. 6 e-mails, that could not be delivered);
- Upper Austria: 42 (excl. 5 e-mails, that could not be delivered);
- Salzburg: 37 (excl. 1 e-mail, that could not be delivered);
- Styria: 50 (excl. 1 e-mail, that could not be delivered);
- Tirol: 40;
- Vorarlberg: 21 (excl. 2 e-mails, that could not be delivered);
- 12 surveys by post (Vienna)

1. The second data collection wave – sending the reminders (total of 429 delivered e-mails + 26 by post):

- Vienna: 145 (excl. 2 e-mails, that could not be delivered);
- Carinthia: 21;
- Burgenland: 7;
- Lower Austria: 64 (excl. 3 e-mails, that could not be delivered);
- Upper Austria: 42;
- Salzburg: 38;
- Styria: 52;
- Tirol: 38;
- Vorarlberg: 22.
- 26 invitations by post (random)

# APPENDIX C

## C1. Descriptive Data of the Dermatologists with a Psychological Background

Table 1

*Socio-demographic Data of the Group of the Dermatologists with a Psychological Background*

| № | Variable | | *n* |
| --- | --- | --- | --- |
| 1. | Gender  Male | *n* = 5 | |
|  | Female | *n* = 6 | |
| 2. | Length of work experience  10 years or less  11-20 years  21-30 years  31 years or more | *n* = 1  *n* = 4  *n* = 5  *n* = 1 | |
| 3. | Contract with an insurance company^a^  Contract physicians  Contract/private dermatologists  Private specialists | *n* = 3  *n* = 3  *n* = 5 | |

*Note*. *N* = 11. *n* = number of participants; Q. – question.

^a^Based on the answers to the Q. 10 “How many patients visits a week do you have?” and Q. 11 “What is the average duration of such visits?”.

On average the group of dermatologists with a psychological background is treating 170.91 patients (*SD =* 133.30) a week. The duration of doctor visits in this group is *M* = 17.05 minutes (*SD =* 8.88).

1. Only two dermatologists have heard something about the SUPRA suicide prevention program. The majority of dermatologists (*n* = 7) knew, that patients with chronic skin conditions are at a higher suicide risk and are suffering more often from suicidal thoughts than a control group. This information they learned primary during training programs (*n* = 6).

Just one dermatologist with a psychological background had more than five suicides in his/her patients for the past year. The rest have chosen the answer “0 or none”. Similar, eight dermatologists had “0 or none” patients with suicide attempts for the past year, three dermatologists chose the answer “I don’t know”. Again eight dermatologists had no patients with suicidal thoughts, three dermatologists had 1-10 such patients for the past year. In case when facing patients at a higher suicide risk, they mostly have a conversation about it (*n* = 9), refer their patients to a specialist in the psychiatry (*n* = 9), or arrange a new appointment (*n* = 6).

The majority of dermatologists with a psychological background (*n* = 6) frequently tells their patients that psychological, psychotherapeutic or psychiatric treatments could be helpful in treating their skin conditions; three doctors are saying it rarely, and two skin doctors are talking about this issue with their patients on all occasions. Five doctors ask their patients frequently about their emotional state, again five physicians do it almost on all occasions, and one doctor asks his or her patients rarely about their emotional state.

To the question, whether dermatologists would have troubles in recognizing suicidal intentions in their patients, six skin doctors chose the answer “rather no”, three doctors said “rather yes”, one dermatologist answered “no”, and again one dermatologist stated “yes, s/he would have troubles to recognize suicidal ideations in his/her patients”. The most challenging about suicide in patients with chronic skin conditions is for the majority of doctors a lack of time (*n* = 4), followed by a lack of knowledge (*n* = 2), suicide-related subjects are not part of their job (*n* = 1), problems in establishing contact with a patient (*n* = 1), and own fear of the emergence of difficult situations that may not be manageable (*n* = 1).

# APPENDIX D: The answers to the open-ended category of survey question

## D1. #21 (“What is the most challenging about suicide in patients with chronic skin conditions?”)

## Original (German)

- Bin offen dafür, stehe aber auf dem Standpunkt dass der Patient das Gespräch darüber suchen soll. (1)
- eine ordentliche Behandlung der Grundkrankheit und eine offene Arzt-Patienten-Beziehung, um die Grundkrankheit unter Kontrolle zu bekommen, dann geht es dem Patienten viel besser (2)
- Ich versuche, die Haut und Befindlichkeit des Pat. zu verbessern, so dass er / sie sich wohl fühlt (2)
- In der Kassensituation ist es kaum möglich, mit dem Dauerzeitmangel so eine Situation zuzulassen. Ich muss 50 Patienten pro 4 Stunden abarbeiten. (4)
- in der Privatordination ist dies keine nicht zu bewältigende Herausforderung (5)
- Kann keine Gedanken lesen (3)
- Motivation des Patienten zum Psychiater (6)
- Suizidalität spezifisch zu erkennen ist im Allgemeinen schwierig (3)
- Wird vom Patienten nicht thematisiert (1)
- Wird von Pat kaum akzeptiert (7)

## Translation (English). Note, as in the original version some sentences are grammatically not correct!

- I’m open to it but from my point of view the patient should seek the conversation. (1)
- Proper treatment of the underlying disease and an open doctor-patient relationship to control the underlying disease, then the patient will feel much better (2)
- I try to improve the skin and condition of the patient, so that he / she feels comfortable (2)
- With the contract health-insurance situation and the lack of time, it is barely possible to allow such a situation. I have to work through 50 patients every 4 hours. (4)
- In the private practice it’s not a non-manageable challenge (5)
- Cannot read minds (3)
- Patient’s motivation to the psychiatrist (6)
- It is generally difficult to identify suicidality specifically (3)
- Doesn’t get addressed by the patient (1)
- Hardly accepted by the patient (7)

## Categories

1. A patient should seek the conversation about it first (*n* = 2).
2. Most important is the proper treatment of the underlying disease and an open doctor-patient relationship (*n* = 2).
3. It is hard to recognize the suicidality in the patients (*n* = 2).
4. Lack of time in a contract practice (*n* = 1).
5. No challenge for a private practice (*n* = 1).
6. Patients’ motivation for a psychiatric treatment (*n* = 1).
7. Is barely accepted by patients (*n* = 1).

## D2. #22 (“How would you evaluate the Picardi et al. (2013) prevention plan?”)

## Original (German)

- Als allgemeine Maßnahme für alle Patienten mit den genannten Erkrankungen nicht sinnvoll. Wie alle anderen diagnostischen Maßnahmen, sollte auch diese nur gezielt und bei klinischem Verdacht/Bedarf eingesetzt werden. Fragebogen ersetzen nicht das Fragen!!! (3.2)
- Aufwendig. (2.2)
- Bei stark auffälligen DLQI Fragebogen (wird routinemäßig erhoben/ dermatologischer Lebensqualitätsindex) Frage ich gezielt nach dem Einfluss der Dermatose auf das psychische Wohlbefinden und je nach Antwort konkret nach Suizidgedanken (4.1)
- Das sollte sich im Rahmen der ärztlichen Beziehung zu PatientInnen ergeben (4.3)
- Eher nicht empfehlenswert. Man könnte damit auch "schlafende Hunde " wecken . (2.4)
- Fragebögen sind in der Praxis ungeeignet. (2.1)
- Fragebogen ja! Ansprechen wäre bei den meisten mit einem nicht zu bewältigendem Zeitaufwand verbunden. (3.3)
- Fragebogen kann ich mir schlecht vorstellen (aber Picardi kann ja gerne in eine typische Kassenordination kommen), die Masse der Patient*Innen leidet nicht so massiv unter den häufig diskreten Hautveränderungen (ist mir aber bekannt, dass Schweregrad und subj. Belastung nicht korrlieren.<br>Wenn muß/müßte ich dies direkt ansprechen.<br>Der letzte Patient mit Suizidgedanken ist wegen eines Heuschnupfen bei mir in Behandlung (3.1)
- fraglicher Aufwand-Nutzen-Relation, da in Klinik und Praxis bisher nie ein Suizid auftrat (2.7)
- gut (1)
- Gut (1)
- gute idee (1)
- gute Idee (1)
- Gute Idee, gutes hilfstool (1)
- guteIdee ! (1)
- hilfreich (1)
- Ich arbeite vor allem mit Allergie Patienten. Es gibt DLQI Bögen für Urtikaria, Asthma, Neurodremitis, Rhinitis, Lebensmittelallergien etc., die im Alltag alle sehr hilfreich wären, aber alle vom Patienten sehr schlecht angenommen werden. Auch solche, die ich im Rahmen von Betreuung von Diplomanden auflege, werden kaum je ausgefüllt. Lange Fragebögen sind völlig unrealistisch in der Praxis. Nicht einmal unser im Qualitätsmanagement aufgelegter Fragebogen mit zwei Fragen wird mehr als zwei Mal pro Woche bei 750 Patienten ausgefüllt (2.6)
- Ich bin mir nicht sicher, ob so ein Fragebogen nicht sogar eine Negativspirale in Gang bringen könnte. (2.4)
- Im Rahmen einer derm Begutachtung kann man natürlich die allgem Befindlichkeit erfragen, jedoch eine Therapieplanung sollte/muß durch den Psychiater erfolgen.<br>Psychologische Maßnahmen wären oft wünschenswert, nur lässt sich eben nicht alles in der Hautsprechstunde abdecken. (3.4, 2.2)
- in der Praxis zu aufwändig, nur im Anlassfall sinnvoll (3.2)
- ist ok (1)
- Könnte sinnvoll sein (1)
- Klingt vernünftig (1)
- mäßig praktikabel (2.1)
- n.a. (4.2)
- nach einem vorhergehenden eingehenden Gespräch willkommen (3.1)
- Nicht bekannt, aber im Psoriasis Komorbiditätsfrsgebogen enthalten (4.2)
- Nicht damit auseinandergesetzt (4.2)
- ok (1)
- Positiv (1)
- praktisches tool zur Wartezeitüberbrückung, aber wie mit dem Ergebnis schließlich umzugehen ist, ist zu hinterfragen. Bei DLQI Befragungen gehe ich auf das Thema der Suizid Gedanken u/o Gefährdung ein und denke, daß dies ausreichend ist. Vorbeugend ist jedenfalls Empathie sicherlich sehr wichtig.... (4.1, 2.3)
- praxisfern (2.7)
- Problem mit der Auswertung/ den patienten darauf ansprechen für dermazologen schwierig, (2.3, 2.5)
- schweirig in der Praxis (2.1)
- Schwierig, da DermatologInnen mit den Antworten selbst sicher nicht umgehen können. (2.3)
- Sehr gut (1)
- sehr gute Idee (1)
- setzen wir gelegentlich ein (4.1)
- sinnvoll (1)
- Wer hat die Zeit? <br>Wer kennt sich aus? (2.2, 4.2)

## Translation (English). Note, as in the original version some sentences are grammatically not correct!

- As a general method for all patients with the named diseases it’s not reasonable. As all other diagnostic measures, it should only be used in a targeted manner and if there is a clinical suspicion/need. Surveys do not replace the asking!!! (3.2)
- Complex (2.2)
- In the case of a highly noticeable DLQI-questionnaire (gets routinely collected/dermatological quality of life index), I specifically ask about the influence of dermatosis on psychological well-being and, depending on the answer, specifically about suicidal thoughts (4.1)
- This should arise in the context of the doctor-patient relationship (4.3)
- Rather not recommended. It could also be used to “stir up a hornet’s nest” (2.4)
- Questionnaires are unsuitable in practice (2.1)
- Questionnaire yes! Talking about it would require an unmanageable amount of time for most people (3.3)
- I can hardly imagine a questionnaire (but Picardi can gladly visit a typical contract practice), most of the patients do not suffer so severely from the often discreet skin changes (but I’m aware that the degree of the severity and the subjective stress do not correlate). If so, I have to/should address this directly. My last patient with suicidal thought is treated by me because of a hay fever. (3.1)
- Questionable cost-benefit ratio since suicide has never occur in clinic and practice (2.7)
- Good (1)
- Good (1)
- good idea (1)
- good idea (1)
- Good idea, good helpful tool (1)
- Good idea! (1)
- Helpful. (1)
- I mainly work with allergy patients. There are DLQI sheets for urticaria, asthma, neurodermitis, rhinitis, food allergies etc., which would all be very helpful in everyday life but are all very poorly accepted by the patient. Even those, that I hand out as part of the supervision of diploma students, are rarely filled out. Long questionnaires are completely unrealistic in practice. Not even our questionnaire with two questions handed out in quality management is filled out more than twice a week in 750 patients. (2.6)
- I am not sure whether such a questionnaire might not even start a negative spiral (2.4)
- As part of a derm assessment, one can of course inquire about the the general condition, but therapy planning should/must be carried out by the psychiatrist. Psychological measures would often be desirable, but you cannot cover everything in the skin consultation hour. (3.4, 2.2)
- too complex in practice, only useful when the need arises (3.2)
- it’s ok (1)
- Might be sensible (1)
- Sounds reasonable (1)
- moderately practicable (2.1)
- N.a. – no answer (4.2)
- welcome after a previous detailed conversation (3.1)
- Not known, but included in the Psoriasis comorbidity questionnaire (4.2)
- Haven’t dealt with it (4.2)
- Ok (1)
- Positive (1)
- practical tool for bridging waiting times, but how to finally deal with the result has to be questioned. In DLQI questionnaires, I address the topic of suicide thoughts and/or danger and I think that is sufficient. As a preventive measure, empathy is certainly very important…(4.1, 2.3)
- unrelated to practice (2.7)
- Problem with the evaluation/ responding to patients difficult for dermatologists (2.3, 2.5)
- hard in practice (2.1)
- Difficult since dermatologists cannot deal with the answer themselves (2.3)
- Very good (1)
- very good idea (1)
- we use it occasionally (4.1)
- usefull (1)
- Who hast the time? Who is well informed? (2.2, 4.2)

## Categories

1. Positive reaction (*n* = 15)
   1. Such as good, helpful, ok.
2. Problems with implementation (problems that can occur during the implementation of the prevention):
   1. moderately practicable (*n* = 3);
   2. too time- or resource consuming (*n* = 3);
   3. problems with the data analysis (*n* = 3);
   4. talking about suicide can have an adverse effect (*n* = 2);
   5. difficulties in discussing this issue with the patients (*n* = 1);
   6. the patients’ poor acceptance of surveys (*n* = 1).
   7. Questionable cost-benefit ratio (*n* = 2).
3. Restriction in implementation (only parts of the prevention plan should/could be implemented):
   1. clinical dialogues are more appropriate than questionnaires (*n* = 2);
   2. only in case of a clinical indication (*n* = 2);
   3. only questionnaires, since clinical dialogues are too time-consuming (*n* = 1);
   4. a therapy must be done by a psychiatrist (*n* = 1).
4. Others (everything not falling into the other three categories):
   1. a similar prevention plan has already (partially) been implemented (*n* = 3);
      1. For example, Dermatology Life Quality Index Questionnaire (Finlay & Khan, 1994);
   2. not familiar with this prevention plan/no opinion (*n* = 4);
   3. this should result out of the doctor-patient relationship (*n* = 1).

## D3. #23 (“Could clinical psychologists or psychotherapists assist you in your work? If yes, how?”)

## Original (German)

- Übernahme der symptomorientierten Gespräche (1.1)
- Überweisung (1.9)
- Akne ist gewiss keine, Psoriasis und Neurodermitis sind nur sehr bedingt psychosomatische Hautkrankheiten im Sinne der Pathogenese bzw. der Therapie<br>Bei hohem Leidensdruck bzw. Problemen der Akzeptanz einer chronischen Dermatose kann jedoch eine psychiatrische psyhologische Intervention sehr hilfreich sein. (2.2, 1.4)
- aktives Zugehen auf den Patienten , psychologisches Gespräch mit dem Patienten (1.1)
- Als Team-Mitglied (1.3)
- Als zusätzliche Anlaufstelle ohne dass es mich 1 Minute mehr Zeit kostet. (3)
- bei Bedarf zur Verfügung stehen; (1.4)
- Bei psyhosomat Hautetkrankungen (1.1)
- Broschüren (1.2)
- Broschüren zum Austeilen und online Seiten mit Informationen und Kontaktmöglichkeiten (Hotline) (1.2)
- Broschüren, Fortbildungen interdisziplinär (1.2, 1.8)
- Broschüren, Selbsthilfegruppen (1.2)
- Depressionen mit Patienten aufarbeiten (1.1)
- durch Übernahme der psychologischen Betreuung/Management (1.1)
- Durch Einsatz bei Bedarf und Patientenwunsch. (1.4)
- Erreichbarkeit im Bedarfsfall (1.4)
- feedback,<br>niedrig schwellige Anlaufstellen für Patienten die Erstberatung durch Spezialisten benötigen (1.5)
- Fragebogen erarbeiten (1.2)
- Gemeinsame Gespräche mit pat (1.1)
- Guter Austausch, rasche Termine auf beiden Seiten (1.6)
- im Spital bei onkologischen Patienten (1.3)
- kontakt (1.1)
- Leider bisher keine guten Erfahrungen mit Psychologen .Wäre oft sehr froh darüber (4)
- Mit dem Patienten sprechen und Zeit verbringen (1.1)
- Mitbehandlung von psychisch auffälligen hautkranken Patienten (1.1)
- Natürlich wäre ein Team Psychotherapeut/Dermatologe ideal. Aber wer bezahlt den Psychotherapeuten? Bezahlen wollen die wenigsten selber. Das muss die Krankenkasse erledigen. Man zahlt ja so viel ein. (1.3, 2.1)
- niedrigschwelliger Zugang zu Therapeut*Innen (1.5)
- Teamarbeit (1.3)
- Teams, z. B. beim Neuodermitis Trainer. Die Schwierigkeit ist, die schlechte Verrechenbarkeit mit den Kassen. (1.3, 2.1)
- Umgang mit bzw Akzeptanz der Erkrankung fördern; <br>teilw Ursachen bei Psychosomatik professionell erfassen und bei Bedarf eine psychiatrischen Begutachtung / Therapie veranlassen<br>besseres Verständnis für die individuellen Bedürfnisse des Patienten aufbauen (1.1)
- Unabhängig von der Dermatolog Behandlung (3)
- v.a. im Spital (1.3)
- Wäre ein tolles Aufgabengebiet in Spezialambulanzen.<br> In einer PrivatPraxis kann man natürlich auch eine Spezialisierung auf Psychosomatik machen, <br>in einer Kassenpraxis wird es wohl mit der Krankenkassenerstattung schwierig werden. (1.7, 2.1)

## Translation (English). Note, as in the original version some sentences are grammatically not correct!

- Taking over the symptom-oriented discussions (1.1)
- Referral (1.9)
- Acne is certainly not, psoriasis and neurodermatitis are only to a very limited degree psychosomatic skin diseases in terms of pathogenesis or therapy. However, psychiatric psychological intervention can be very helpful if there is a high level of suffering or problems with the acceptance of chronic dermatosis (2.2, 1.4)
- active approach to the patient, psychological discussion with the patient (1.1)
- As a team member (1.3)
- As an additional point of contact without costing me 1 minute more of time (3)
- be available when needed (1.4)
- For psychosomat skin diseases (1.1)
- Brochures (1.2)
- Brochures to hand out and online pages with information and contact options (hotline) (1.2)
- Brochures, further training interdisciplinary (1.2, 1.8)
- Brochures, self-help groups (1.2)
- Work through depressions with patients (1.1.)
- by taking over psychological support / management (1.1)
- By use when needed and patient request (1.4)
- Availability when needed (1.4)
- feedback, low-treshold contact points for patients who need initial advice from specialists (1.5)
- Develop a questionnaire (1.2)
- Joint conversations with patient (1.1)
- Good exchange, quick appointments on both sides (1.6)
- in hospital by oncological patients (1.3)
- contact (1.1)
- Unfortunately, no good experiences with psychologists so far. I often would be very happy about it (4)
- Talk to the patient and spend time (1.1)
- Co-treatment of psychologically noticeable skin sick patients (1.1)
- Of course, a team of psychotherapist/dermatologist would be ideal. But who pays the psychotherapist? Very few want to pay themselves. The health insurance company has to do that. One pay in so much (1.3, 2.1)
- low-threshold access to therapists (1.5)
- Team-work (1.3)
- Teams, e.g. at the neurodermatitis trainer. The difficulty is the poor billing with the health insurance companies. (1.3, 2.1)
- Promote dealing with or acceptance of the disease. In cases of psychosomatics, professionally record causes and, if necessary, arrange psychiatric assessment/therapy. develop a better understanding of the individual needs of the patient (1.1)
- Independent from the dermatologic treatment (3)
- especially in the hospital (1.3)
- Would be a great area of responsibility in dedicated outpatient clinics. In a private practice you can of course also specialize in psychosomatic medicine, in a contract practice, health insurance reimbursement will probably be difficult (1.7, 2.1)

## Categories

1. Expected help from the side of mental health professionals:
   1. by taking over the psychological care of patients (*n* = 10), such as
      1. symptom-oriented dialogues, treatment of depressions, facilitation of the acceptance of the disease;
      2. professionally identify the causes of psychosomatic disorders;
      3. promote a better understanding of patients’ individual needs.
   2. by developing questionnaires, brochures, websites, hotlines, support groups, etc. (*n* = 5);
   3. as a member of a team, especially in a hospital (*n* = 6);
   4. availability in a case of need (*n* = 4);
   5. low-threshold psychological treatment (*n* = 2);
   6. exchange of experience, quick appointments on both sides (*n* = 1);
   7. in specialized outpatient clinics or private practice (*n* = 1);
   8. interdisciplinary trainings (*n* = 1);
   9. doctor’s referral (*n* = 1).
2. Problems with implementation:
   1. the difficulty with health insurance reimbursement, especially in contract physician practices (*n* = 3);
   2. these skin conditions do not really belong to the group of psychosomatic skin disorders in terms of pathogenesis or therapy (*n* = 1).
3. Restrictions in implementation: as an additional treatment place that runs independently from a dermatologist practice (*n* = 2).
4. Others: no good experiences with psychologists (*n* = 1).
